# Vaccine with bacterium‐like particles displaying HIV‐1 gp120 trimer elicits specific mucosal responses and neutralizing antibodies in rhesus macaques

**DOI:** 10.1111/1751-7915.14022

**Published:** 2022-03-15

**Authors:** Huaiyu Wang, Pingchao Li, Mo Zhang, Jinpeng Bi, Yizi He, Fangshen Li, Rongzhen Yu, Feng Gao, Wei Kong, Bin Yu, Ling Chen, Xianghui Yu

**Affiliations:** ^1^ National Engineering Laboratory for AIDS Vaccine School of Life Sciences Jilin University Changchun 130012 China; ^2^ State Key Laboratory of Respiratory Disease Guangzhou Institutes of Biomedicine and Health (GIBH) Chinese Academy of Sciences Guangzhou 510530 China; ^3^ University of Chinese Academy of Sciences Beijing 100049 China; ^4^ Department of Medicine Duke University Medical Center Durham NC 27710 USA; ^5^ Key Laboratory for Molecular Enzymology and Engineering, The Ministry of Education, School of Life Sciences, Jilin University Changchun 130012 China; ^6^ The First Affiliated Hospital of Guangzhou Medical University Guangzhou 510060 China

## Abstract

Preclinical studies have shown that the induction of secretory IgA (sIgA) in mucosa and neutralizing antibodies (NAbs) in sera is essential for designing vaccines that can effectively block the transmission of HIV‐1. We previously showed that a vaccine consisting of bacterium‐like particles (BLPs) displaying Protan‐gp120AE‐MTQ (PAM) could induce mucosal immune responses through intranasal (IN) immunization in mice and NAbs through intramuscular (IM) immunization in guinea pigs. Here, we evaluated the ability of this vaccine BLP‐PAM to elicit HIV‐1‐specific mucosal and systemic immune responses through IN and IM immunization combination strategies in rhesus macaques. First, the morphology, antigenicity and epitope accessibility of the vaccine were analysed by transmission electron microscopy, bio‐layer interferometry and ELISA. In BLP‐PAM‐immunized macaques, HIV‐1‐specific sIgA were rapidly induced through IN immunization in situ and distant mucosal sites, although the immune responses are relatively weak. Furthermore, the HIV‐1‐specific IgG and IgA antibody levels in mucosal secretions were enhanced and maintained, while production of serum NAbs against heterologous HIV‐1 tier 1 and 2 pseudoviruses was elicited after IM boost. Additionally, situ mucosal responses and systemic T cell immune responses were improved by rAd2‐gp120AE boost immunization via the IN and IM routes. These results suggested that BLP‐based delivery in combination with the IN and IM immunization approach represents a potential vaccine strategy against HIV‐1.

## Graphical abstract

PAM bound to BLPs effectively and the antigenicity, epitopes accessibility was kept well. Intranasal immunization of BLP‐PAM rapidly induced HIV‐1‐specific sIgA in multiple mucosal tissues and moderate systemic immune response in rhesus macaques. Intramuscular immunization of BLP‐PAM induced NAbs in sera and enhanced the level of IgG in mucosal tissues.

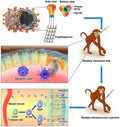

## Introduction

HIV‐1 is the primary AIDS‐causing pathogen. Approximately 38.0 million people globally were living with HIV, while 1.7 million people became newly infected with HIV, in 2019 (data from aidsinfo.unaids.org). The drug resistance of the virus and the number of HIV positive patients put high pressure on antiretroviral therapy. However, an effective vaccine design represents the best approach to prevent HIV from spreading.

HIV transmission takes place mainly through mucosa during sexual intercourse. While more than 85% of lymphoid tissue and nearly 90% of lymphocytes are distributed in mucosa, especially in gastrointestinal tract, and a significant depletion of CD4^+^ T cells was observed at all stages of HIV, the immune dysfunction and the loss of intestinal barrier function are correlated to the deletion of CD4^+^ T cells in gut‐associated lymphoid tissue, which are early targets of HIV‐1 and potential early reservoirs for the replication sites (Lim, 1993; Allers *et al*., 2016). The vaccination that intends to build the first defence barrier at mucosa is expected to interrupt the HIV‐1 infection to mucosal Langerhans cells, dendritic cells and macrophages, thus prevent the body from HIV‐1 infection. Clinical researches also provided some clues for mucosal vaccination. HIV‐specific secretory IgA (sIgA) and IgG were detected in the cervico‐vaginal secretions of HIV‐exposed uninfected individuals (Devito *et al*., 2000). Furthermore, IgA purified from the mucosa of persistently seronegative individuals was able to inhibit HIV‐1 transcytosis. Secretory IgA responses are associated with the resistance against HIV‐1 acquisition (Benjelloun *et al*., 2013; Taha Hirboda *et al*., 2018), which can contribute to immune viral exclusion, inhibited transcytosis and intracellularly neutralization of HIV‐1 before it establishes a reservoir and produces viral copies, as seen in chronically infected hosts (Devito *et al*., 2000; Wright *et al*., 2008).

In addition to the mucosal barrier, serum neutralizing antibodies (NAbs) are necessary to inhibit the spread of HIV in the body. Due to the HIV‐1 diversity, the use of broadly reactive NAbs (bNAbs) against the HIV‐1 envelope glycoprotein (Env) trimer is recommended for an effective vaccine (Steichen *et al*., 2019). A major challenge in inducing such bNAbs through vaccination is represented by the need to design stable soluble mimics of the native‐like trimer immunogens, such as BG505 SOSIP.664, NFL trimer and UFO trimer (Andrabi *et al*., 2018). Displaying these trimer antigens in a particular array on synthetic nanoparticles (NPs) or self‐assembling nanoparticle is an established strategy for developing high‐affinity B cell receptors (BCRs) and ultimately generating highly neutralizing antibody responses (Dubrovskaya *et al*., 2019; Sliepen *et al*., 2019). For instance, multivalent antigens delivered via micro/nanocarriers that can mimic the repetitive and well‐ordered antigenic structures cross‐link BCRs and activate B cells more efficiently than their monovalent counterparts.

To induce simultaneous HIV‐1‐specific mucosal and systemic immune responses, the use of two different vaccine formats (a viral vector vaccine and protein vaccine combined immunization) is often needed (Kang *et al*., 2018; Jones *et al*., 2019). Currently, there is rarely a single vaccine have been reported that can induce both mucosal responses and serum NAbs via the conventional immunization route. Protein vaccines with ideal carriers or adjuvants represent good candidates for achieving this goal (Morgane *et al*., 2011; Arunachalam *et al*., 2020). Previously, we found that gp120 trimers bound to bacterium‐like particles (BLPs) based on non‐recombinant *Lactococcus lactis* bacteria delivered via intranasal (IN) drip successfully induced Env‐specific sIgA at multiple mucosal sites in mice. Furthermore, this vaccine‐induced strong neutralizing antibodies in guinea pigs via intramuscular (IM) injection (Bi *et al*., 2020). In this study, we further investigated the immunogenicity of an IN prime/IM boost HIV‐1 BLP‐based vaccine in rhesus macaques. Although a variety of BLP‐based vaccines such as influenza and RSV vaccines can effectively induce mucosal immune responses in mice, currently, the immunogenicity of these vaccines has not been assessed in non‐human primates (Van Braeckel‐Budimir *et al*., 2013).

## Results

### Biophysical and biochemical analysis of the gp120 trimers

**Fig. 1:**
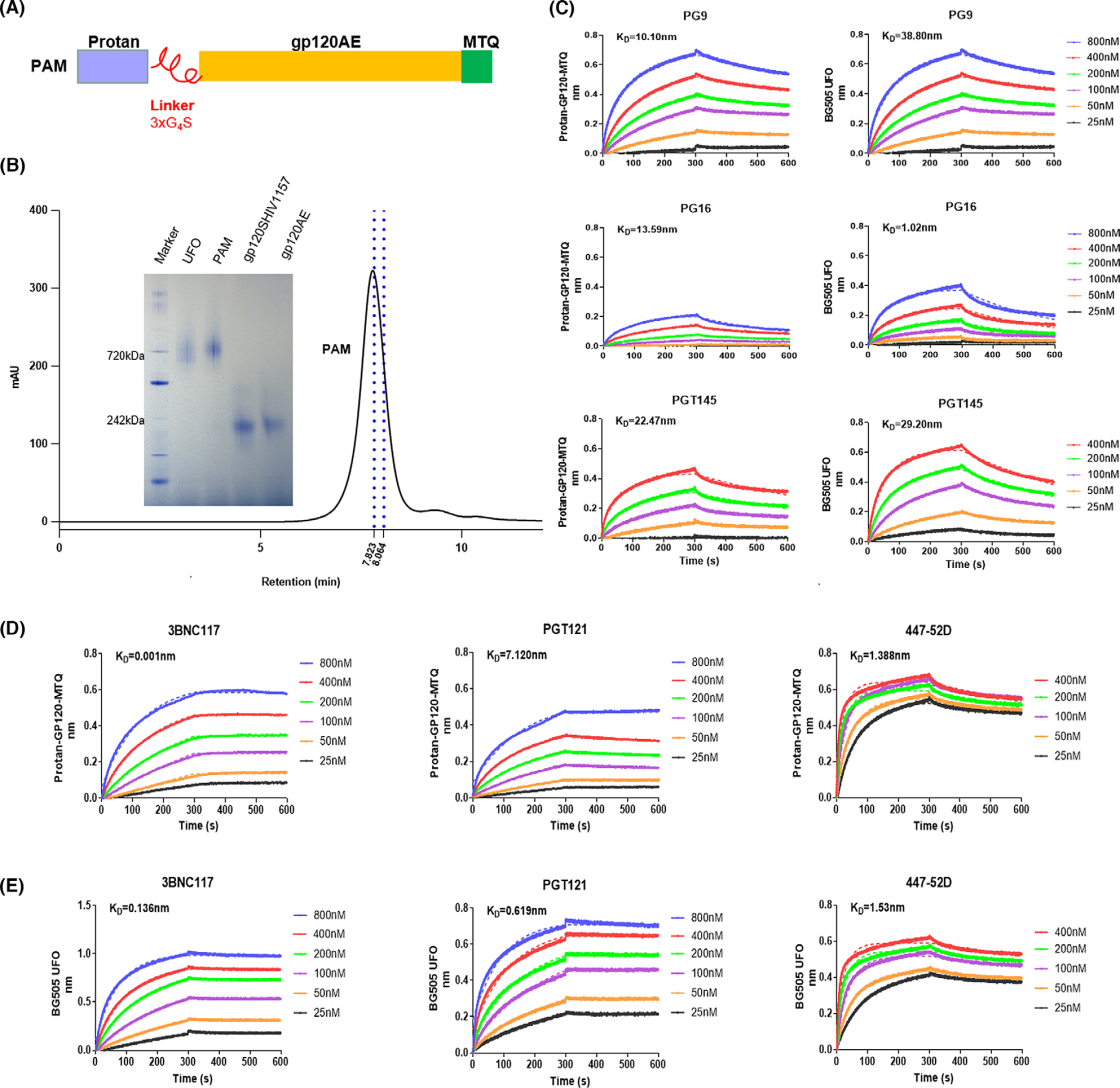
Biophysical and biochemical characterization of PAM trimers by HPLC and Bio‐layer interferometry. A. Schematic representation of PAM. B. Native PAGE and HPLC analyses of PAM compared with UFO trimer and gp120 monomer antigens. HPLC retention time of PAM are shown in black, while the blue lines indicate the molecular mass markers (from left to right: 669 and 440 kDa). C–E. Structure and antigenic profiles of PAM trimers and BG505 UFO measured against a panel of representative bNAbs and non‐NAbs using Octet RED96 and a trimer titration series of six concentrations. The first 300 s illustrate the association process, while the 300–600 s interval illustrates the dissociation process.

The gp120 trimer (PAM) was based on a transmitted/founder virus (BJOX015000.11.5) *env* gene from CRF01_AE identified in HIV‐1‐infected MSM individuals in Beijing, China. We added Protan at the N‐terminal of gp120 to aid in BLP binding, and we added a trimerization motif (MTQ) to replace gp41 at the C‐terminal for stabilizing the trimer and reducing the dissociation effect induced by furin cleavage and to minimize the exposure of immunodominant non‐neutralization epitopes that might induce base‐specific mAbs such as 12N (Fig. 1A) (Moyer *et al*., 2020). The molecular weight of PAM trimers was analysed using native polyacrylamide gel electrophoresis (PAGE) with BG505 UFO (a native‐like trimer), SHIV1157 gp120 and HIV‐1 gp120AE monomer (used as a control). We showed that the PAM trimer and gp120 monomer were over 720 kDa and close to 242 kDa respectively. The PAM trimers were larger than BG505 UFO due to the addition of Protan (Fig. 1B). The purity of PAM trimers was further examined using HPLC. The peak retention time of PAM was 7.808 min, which was shorter than that of BG505 UFO (8.019 min) and similar to that of Ferritin (7.823 min), which had a molecular weight of 660 kDa (Fig. S1A–C).

To further evaluate the conformation and epitope exposure of PAM, several representative NAbs were used to test the binding with PAM trimer using Bio‐layer interferometry. In this assay, the binding of mAbs to the PAM trimer was assessed during the moving phase (shaking of the plates at 1000 rpm/min), so that the association and disassociation process could be monitored, unlike following ELISA. NAbs were mainly categorized into five major groups, based on the dominant target sites (Zhou and Xu, 2018). Due to the use of the gp120 trimer antigen, we chose 3BNC117 targeting CD4‐binding site, PG9 targeting V1/V2 apex, as well as PGT121 and 447‐52D (non‐NAbs) targeting V3‐glycan. First, the trimer could intermediately bind to PG9, PG16 and PGT145. Since PG9, PG16 and PGT145 were quaternary structure‐dependent (Julien *et al*., 2013), these results indicated that PAM exhibited a steric conformation and closed to native‐like trimers (Fig. 1C). Compared with the binding of BG505 UFO to these three mAbs, we found that the binding capacity of PAM and PG16 is weaker than BG505 UFO and PG16, indicating that the trimeric structure of PAM is different from BG505 UFO. For the epitope exposure analysis, PAM trimers bound strongly to 3BNC117, which indicates that the epitopes of CD4‐binding site were well exposed on PAMs (Fig. 1D). PG9 was also a N160 glycan‐specific bNAb, while PGT121 was a N332‐dependent bNAb; thus, the intermediate binding of PAM to PG9 and PGT121 showed that the glycan modification of PAM at these sites were also exposed (Fig. 1D). The 447‐52D antibody, which mainly targeted the GPGQ motif at the tip of the V3 loop, interacted with PAMs containing the GPGQ motif, indicating the exposure of this motif (Fig. 1D). The fast association and intermediate dissociation suggest that the V3 loop structure of PAM might be affected by complicated glycan modification or glycan interaction (Ward and Wilson, 2015). *K*_on_ and *K*_off_ values are listed in Table S1. In addition, the binding capacity of PAM and PG16, 3BNC117 and PGT121 is obviously different from BG505 UFO and these three mAbs, indicating that the exposed epitopes of PAM are also different from BG505 UFO (Fig. 1C–E). These results showed that PAM had great purity and was a mimic of native‐like trimer, based on the molecular weight and epitopes exposure analysis.

### BLPs effectively present PAM trimers without masking antigenic epitopes

**Fig. 2:**
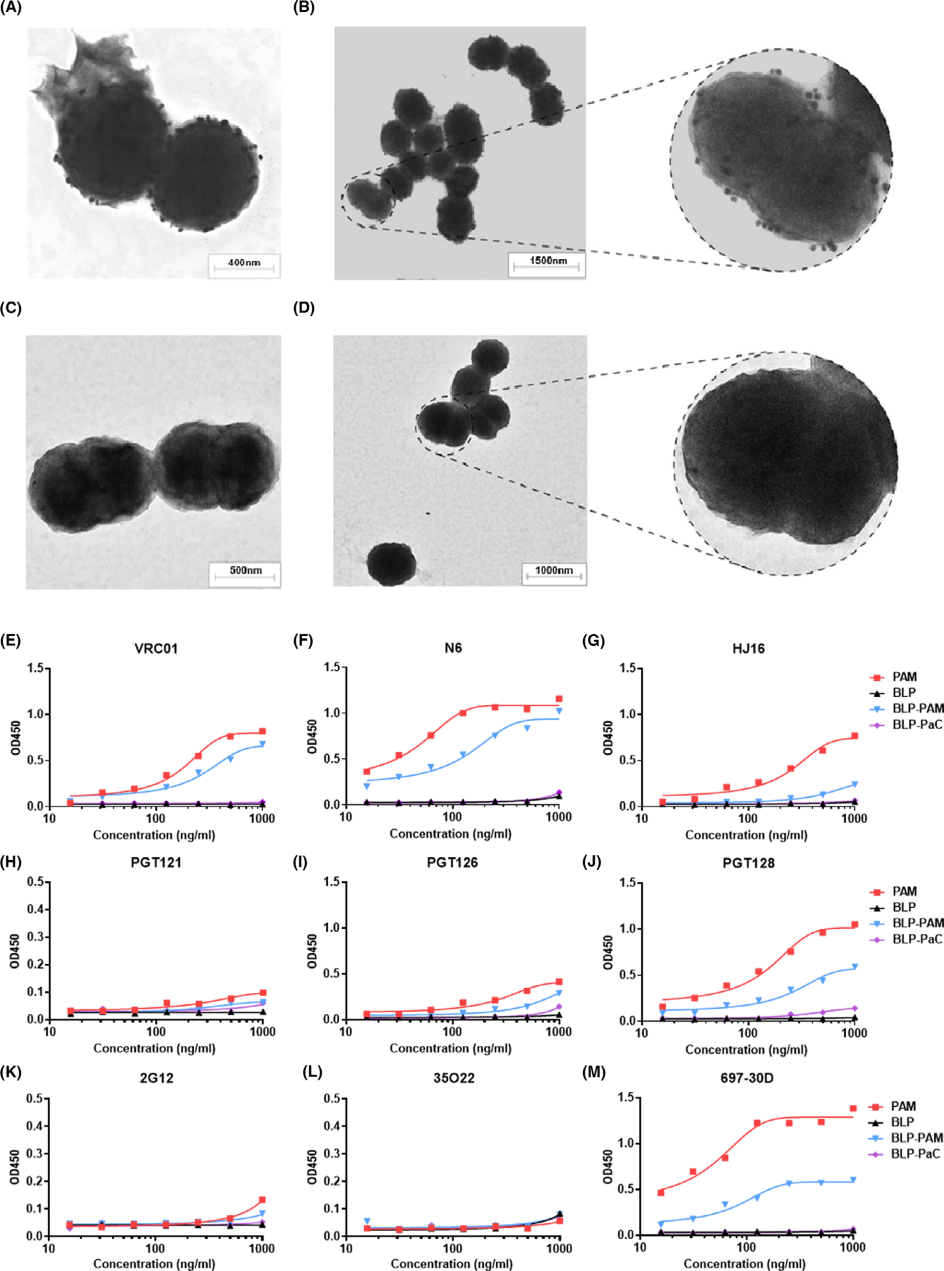
Analysis of PAM binding to BLPs and the antigenic analysis of BLP‐bound PAM trimers. A, B. Negative stain TEM images of BLP‐PAM coated with VRC01 and labelled using 30 nm immunogold goat anti‐human IgG. C, D. Negative stain TEM images of BLPs coated with VRC01 and labelled using immunogold goat anti‐human IgG. All images are at 12 000× magnification. E–G. Representative bNAbs targeting CD4bs. H–J. Representative bNAbs targeting the V3 region. K–M. Representative bNAbs targeting an OD‐glycan (2G12), the gp120‐gp41 interface (35O22) and V2 (697‐30D) respectively. Blank BLP and BLP‐PaC (norovirus P particles‐bound BLPs) were used as controls.

Previously, using Coomassie Blue Staining and immunofluorescence assay, we demonstrated that Protan‐fused gp120 can bind to BLP and that 30 μg of antigen bound to 1 mg of BLP (Bi *et al*., 2020). To intuitively and clearly examine the binding of antigens to BLPs, we performed negative staining of the trimer‐coupled BLPs followed by TEM imaging. As the diameter of BLP was approximately 1 μm and vastly larger than PAM, 30 nm immunogold labelled goat anti‐human IgG was used to visualize the binding. The results demonstrated that the PAM trimers bound the BLP surface effectively and evenly, and the BLP morphology following protein binding was not changed compared with that of the negative control (Fig. 2A–D, Fig. S2A,B). Additionally, these results indicated that PAM could bind to the BLP surface in a targeted manner, without affecting the exposure of the epitope, as we used the monoclonal antibody VRC01 to indirectly confirm whether PAM binds to BLPs.

To further evaluate whether multiple neutralizing epitopes on PAM trimer were blocked by BLP following non‐covalent binding, ELISA was used to analyse the antigenicity of PAM displayed on BLPs using a large panel of representative bNAbs and non‐NAbs. The antigenicity was analysed using mAbs including the NAbs targeting the CD4‐binding site (VRC01, N6, HJ16, 3BNC117 and VRC‐CH31), V1V2 (PG9 and PGT145), V3‐glycan (PGT121, PGT126, PGT128 and 10‐1074), linear epitope (697‐30D), gp120‐gp41 interface (35O22) and OD‐glycan (2G12). PG9 and PGT145, which span the V1V2 domains of two gp120 subunits within one trimer, were usually used to evaluate whether soluble Env trimers adopt the proper conformation (Julien *et al*., 2013).

For CD4‐binding site epitopes, the ELISA results indicated that the PAM trimer was efficiently recognized by VRC01, N6, HJ16 and 3BNC117; however, the binding to VRC‐CH31 was weak and showed non‐reactivity for non‐NAbs F105, which suggests that the epitopes targeted by F105 were undetectable on PAM (Fig. 2E–G, Fig. S3A–C). For V1V2 quaternary epitopes, PG9 bound strongly to PAM, while the binding to PGT145 and PG16 was weak (Fig. S3D–F). For V3‐glycan, PGT121 (which is conformationally dependent on the N332 glycan), PGT126, PGT128 and 10‐1074‐like antibodies recognizing V3 were chosen (Walker *et al*., 2011). The observed binding of PAM to PGT121 was weak, while the binding to 10‐1074 and PGT126 was intermediate (Fig. 2H–J, Fig. S3G). Furthermore, PAM showed high affinity for PGT128 and 697‐30D, which target the V2 region of gp120 164‐194 (HXB2) and are conformation‐dependent (Fig. 2M) (Gorny *et al*., 1994; Walker *et al*., 2009). In contrast, PAM did not bind to some non‐NAbs (F105) and NAbs targeting the gp120‐gp41 interface (35O22). Overall, BLP‐PAM and PAM exhibit approximately the same binding abilities to antibodies against different epitopes.

All NAbs and non‐NAbs, which bound to BLP‐PAM were not able to bind to BLP. Due to the limitation of the coating process, some protein trimers connected to BLP were compressed when binding to the plates; thus, our experiments could only prove that the trimer protein was effectively connected to BLP, and the PAM epitopes of PAM were not hidden‐masked or blocked by BLP. Even though we were able to identify the clear difference in PG9, HJ16 and 3BNC117, we determined that a variety of neutralizing sites recognized by bNAbs on the PAM surface were accessible after binding to BLP, which is in line with a previous study. Here, we used blank BLP and an unrelated protein bound to BLP as negative controls to ensure that the used mAbs have no non‐specific binding to BLP. These results demonstrated that PAM can effectively bind to BLPs, and the antigenicity and epitope accessibility were maintained.

### Rapid induction of sIgA via BLP‐PAM intranasal immunization

**Fig. 3:**
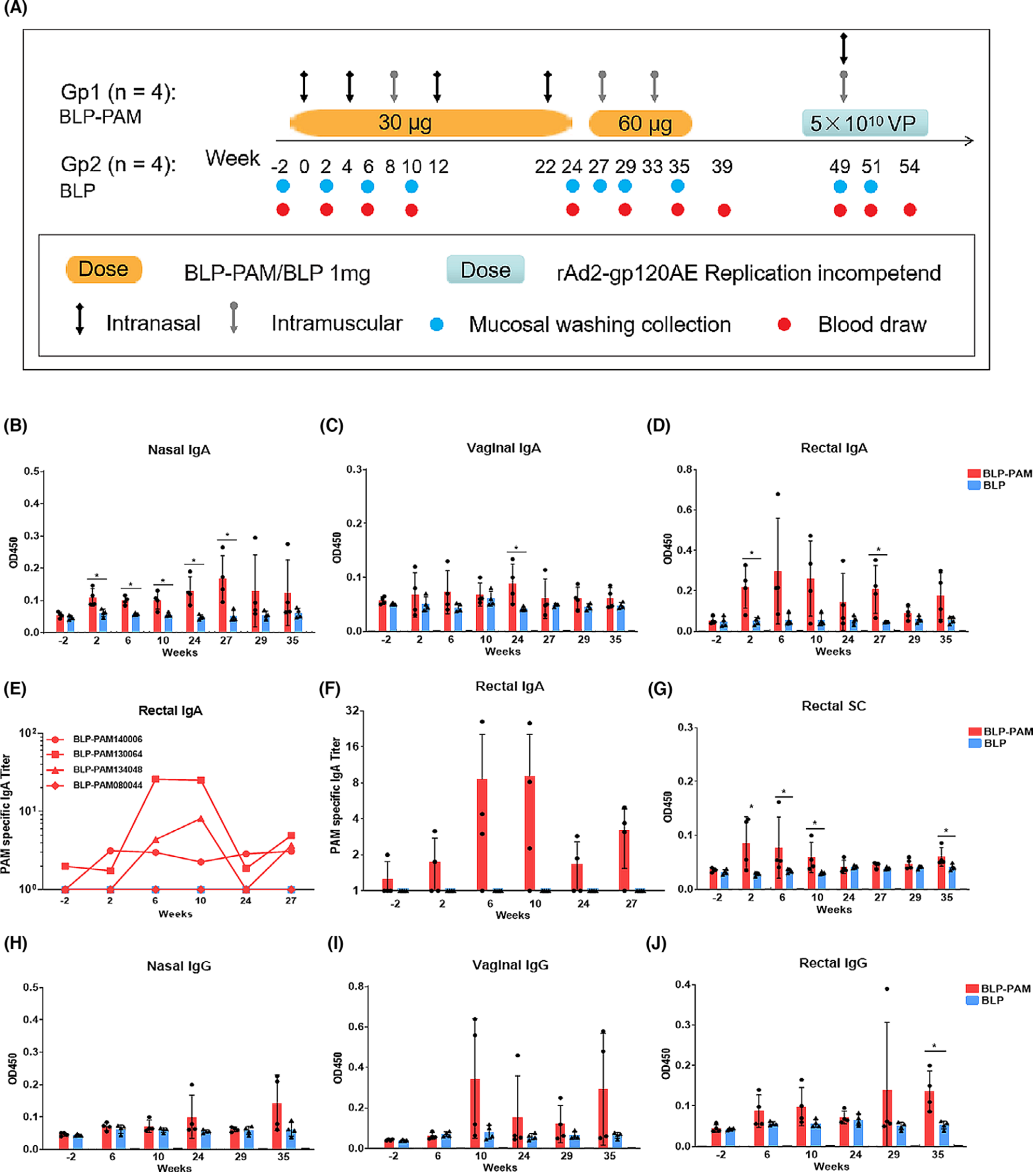
Immunization schedule and the mucosal immune responses induced by BLP‐PAM in rhesus macaques. A. Timeline of BLP, BLP‐PAM and rAd2‐gp120AE immunization and sample collection. Four macaques in each group were immunized with BLP or BLP‐PAM via an IN or IM route at different time points using the doses indicated in the Materials and Methods section. For the adenoviral vector vaccine rAd2‐gp120AE, two groups of macaques were simultaneously immunized at week 49 with the same dose of vaccine via both IN and IM routes. B–D. IgA antibody levels in nasal, vaginal and rectal washings. E, F. End‐point titres of IgA antibody levels in rectal samples obtained from individual macaques at the indicated time points. G. SC levels in rectal washings measured using ELISA from samples collected at the indicated time points. H–J. IgG antibody levels in nasal, vaginal and rectal washings. Mean and standard deviation are indicated with lines and error bars, *P < 0.05.

The transmission of HIV takes place mainly through mucosal tissues, while the sIgA and IgG from the mucosa can block the HIV transcytosis through epithelial cells (Pavot *et al*., 2014). We previously showed that BLP‐PAM could induce sIgA in mucosal samples through IN immunization in mice and induce strong IgG and NAbs in serum through IM immunization in guinea pigs (Bi *et al*., 2020). Therefore, we designed combining IN and IM routes of immunization to investigate whether it can induce mucosal responses and neutralizing antibodies in non‐human primate model, since this model is more suitable for evaluating these two kinds of immune responses together. The basic design is two IN immunizations followed by one or two IM immunizations and then boost with BLP‐PAM or an adenovirus vector vaccine according to the strength of the immune response. However, the fourth sample collection was skipped, and the fifth immunization was delayed due to the restrictive effects of the COVID‐19 pandemic on the research practice. The final immunization schedule, doses used, and sample collections are summarized in Fig. 3A. Two groups of rhesus macaques were immunized with BLP‐PAM and BLP via an IN route at 0, 4, 12 and 22 weeks followed by IM immunizations at 8, 27 and 33 weeks. The mucosal washing and plasma samples were collected two weeks after each immunization.

HIV‐1 Env‐specific IgA antibodies were measured using ELISA and were detectable after the first IN drip in nasal, rectal and vaginal samples from at least one macaque within BLP‐PAM group, while no IgA antibodies were observed in the ‘empty’ BLP group. In nasal samples, the IgA levels of different macaques in the experimental group were stable and significantly higher than control group from 2 weeks to 27 weeks and further enhanced during the last IN immunization. The nasal IgA level reached the peak at 27 weeks, before the sixth immunization (Fig. 3B–D). The IgA level in rectal samples was higher than those from nasal and vaginal samples, which was enough to be calculated for titre (Fig. 3E–F). The IgA antibody levels in the vaginal samples were the lowest among the different sampling locations and vastly different among individual macaques (Fig. 3C). We also found that IM immunizations did not increase the IgA antibody levels in nasal, rectal and vaginal secretions.

To further verify whether the increase in mucosal IgA levels produced sIgA, we measured the level of secretory component (SC), which is a part of secretory IgA (Ruprecht *et al*., 2019). Lamina propria plasma cells produce dimerized IgA, which use pIgR passing through epithelial cells to generate sIgA. The SC levels of rectal washing samples and week 27 nasal samples from BLP‐PAM group could be measured and the BLP‐PAM group showed significantly higher than BLP group at 6,10 and 35 weeks (Fig. 3G). However, the SC levels in other nasal and vagina samples were not detectable due to the low IgA levels (Fig. S4A–B). Nevertheless, these results suggested that BLP displaying PAM was able to rapidly induce sIgA in different mucosal tissues through IN immunization.

Due to sampling limitations, we only assessed the HIV‐1‐specific IgG level in mucosal samples collected on weeks −2, 6, 10, 24, 29 and 35. The IgG level was increased following IM immunization in all mucosal samples. The IgG levels in vaginal samples were noticeably higher than those observed in rectal and nasal samples. Furthermore, a strong IgG response following the first low‐dose IM injection was detected in vaginal samples from two macaques in the BLP‐PAM group (Fig. 3H–J). Thus, in rhesus macaques, IN immunization of BLP‐PAM could rapidly induce HIV‐1 Env‐specific mucosal immune responses in local and distal mucosal tissues, while the subsequent IM immunization boosts maintained the sIgA level and enhanced mucosal IgG antibody levels, especially in vaginal secretions.

### Intramuscular boost immunization of BLP‐PAM increases the systemic humoral immune response

**Fig. 4:**
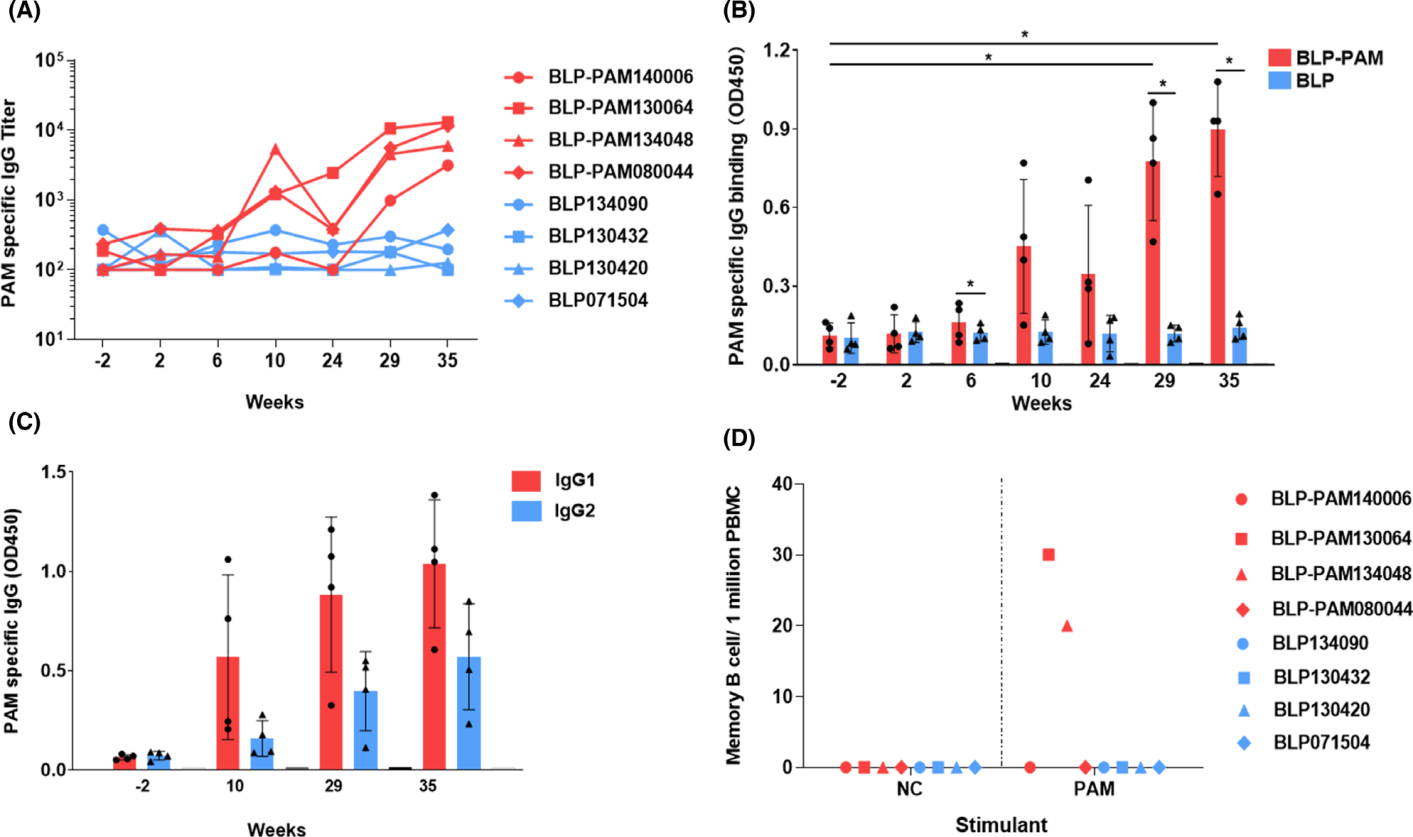
Analysis of B cell responses induced by BLP‐PAM in blood. A. Serum IgG antibodies titres obtained from individual macaques at the indicated time points. B. Serum IgG antibodies level obtained from macaques in group at the indicated time points. C. BLP‐PAM serum IgG isotype analysis at the indicated time points. D. PAM‐specific IgG memory B cells among PBMCs measured using B cell ELISPOT assay at week 39. Mean and standard deviation are indicated with lines and error bars, *P < 0.05.

An HIV vaccine should provide protection for both mucosal surface and the adaptive immune system (Demberg and Robert‐Guroff, 2009). To evaluate the systemic immune response, we measured HIV‐1 Env‐specific IgA and IgG levels in the serum of macaques. Compared with the BLP control group, the BLP‐PAM group showed low levels of IgG antibodies after the second IN immunization at week 6. In contrast, a single IM immunization boost increased serum PAM‐specific IgG titres by approximately 10‐fold compared with the first two IN immunizations. Furthermore, the serum IgG titres reached a significant value after the third IM immunization with the increased antigen dose (Fig. 4A,B) and the specific IgG titres were significantly higher in BLP‐PAM group compared with BLP group since 29 weeks (Fig. 4B). While analysing the phenotype of the immune responses following IM boost immunization, we found that both IgG1 and IgG2 were produced in macaques, wherein the IgG1:IgG2 ratio was relatively low, thereby indicating that the antibody response induced by BLP‐PAM immunization exhibits a balanced Th1/Th2 phenotype (Fig. 4C, Fig. S5A–E).

To further evaluate the persistence of the HIV‐1 Env‐specific responses generated by BLP‐PAM, circulating PAM‐specific antibody‐secreting cells (ASCs) were quantified 6 weeks after the last immunization, following an in vitro stimulation meant to enumerate antigen‐specific memory B cells. As shown in Figure 4D, two macaques from the BLP‐PAM group had a moderate number of circulating IgG ASCs. To analyse the T cell immune responses of BLP‐PAM, we used three HIV‐1 clade CRF‐01 AE Env peptide pools of PBMCs and measured the Env‐specific T lymphocyte responses using IFN‐γ ELISPOT assays (Fig. S6A). At week 39, no positive cell was observed in any of the immunized macaques. Furthermore, IL‐2‐, TNF‐α‐ and IFN‐γ‐producing CD4^+^ T cells were undetectable among PBMCs measured using fluorescence‐activated cell sorting (Fig. S6B–D).

Collectively, these data demonstrated that a combined IN and IM immunization with BLP‐PAM might induce strong systemic B cell immune responses rather than T cell immune responses.

### BLP‐PAM vaccination induces neutralizing antibodies in blood

**Fig. 5:**
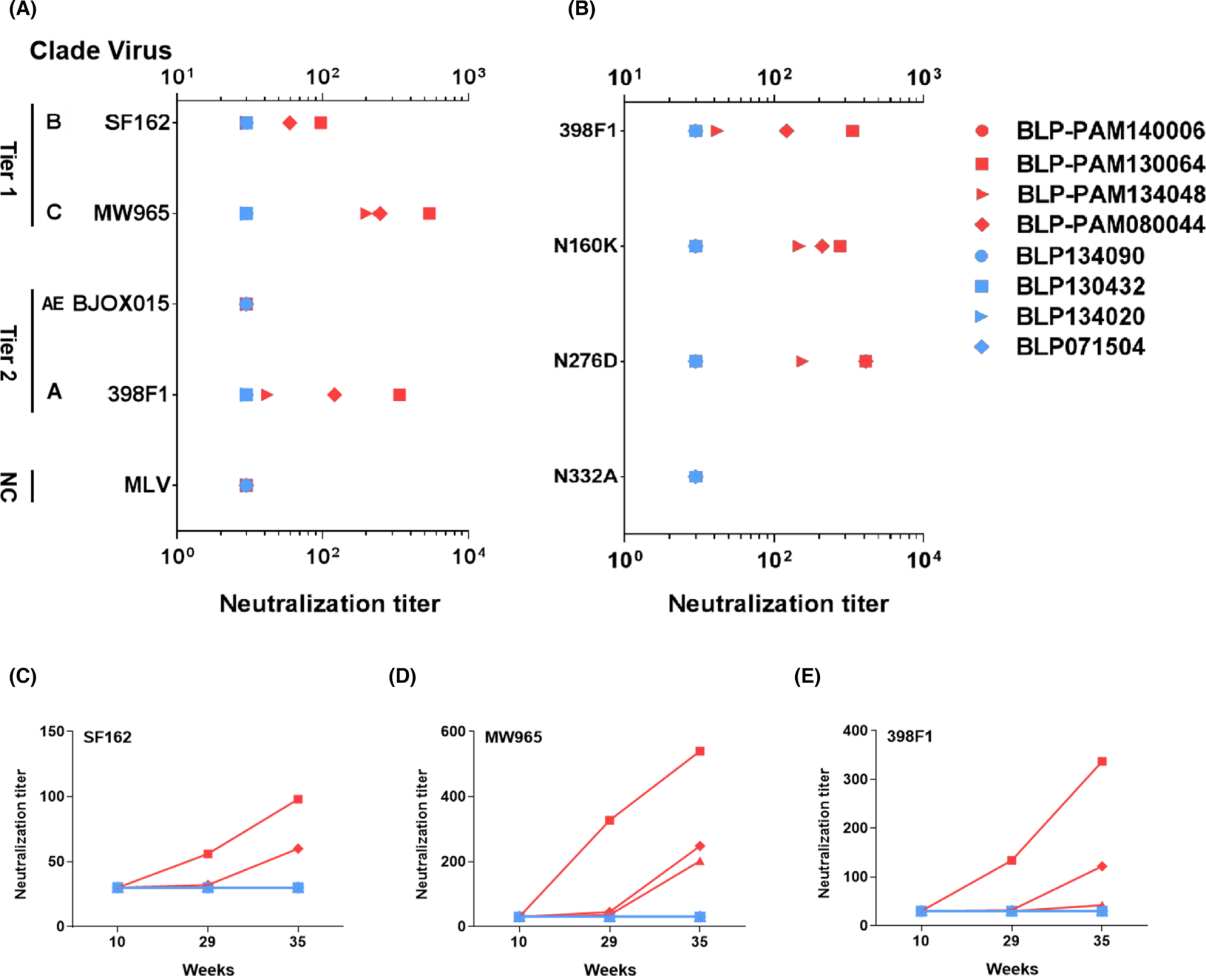
Neutralization titres of sera from rhesus macaques tested against a panel of Env‐pseudotyped viruses and mutants. A. Neutralization titres of macaque sera samples (week 35) against representative tier 1 and tier 2 viruses. MLV was used as the negative control. B. Neutralization titres from macaque sera samples at week 35, against a panel of 398F1 virus mutants. C–E. PAM‐BLP 3rd, 5th and 7th sera neutralization potency against SF162, MW965 and 398F1 viruses.

Broadly reactive NAbs can be found in plasma of infected individuals exposed to HIV for several years. The passive transfer of bNAbs has been shown to provide macaques with a complete protection against mucosal challenge (Pegu *et al*., 2017). Inducing bNAbs in a short period of time with manageable number of immunogens represents an ideal characteristic a vaccine should exhibit. TZM‐bl neutralization assays were used to assess the development of NAbs two weeks after each IM immunization. Neutralization breath was assessed using 12‐virus panel of tier 2 isolates, autologous pseudovirus and 3‐virus of tier 1 isolates. At week 29, 3 out of 4 macaques from the BLP‐PAM group developed heterologous NAb activity (ID_50_ titre > 30) against the tier 1 clade C virus MW965, while one monkey exhibited cross‐neutralizing activity against tier 1 clade B virus SF162 and tier 2 clade A virus 398F1. After the third IM immunization (week 35), the other two macaques also developed cross‐neutralizing activity and the NAbs titres against MW965, SF162 and 398F1 were increased in all three macaques. However, no NAbs against the autologous virus tier 2 clade AE BJOX015 were detected in any of the macaques at week 35 because the pseudovirus BJOX015 is a neutralization‐insensitive virus (Fig. 5A, Fig. S7A). In contrast, none of the four macaques from the BLP group developed HIV‐1‐specific NAbs (Fig. S7B).

Further, we evaluated the sera NAb activity against a small panel of 398F1 virus mutants including V2‐glycan N160K, CD4bs N276D and V3‐glycan N332A. The N332A substitution completely abrogated 398F1 neutralization, while the N160K and N276D substitutions consistently enhanced 398F1 sensitivity in two out of three sera by approximately twofold and fourfold respectively (Fig. 5B, Fig. S8). It has been reported that removing the glycan at position 332 can completely abolish the neutralization induced by the PGT121‐123 monoclonal antibodies (Walker *et al*., 2011; Mouquet *et al*., 2012). These results indicated that the NAbs against 398F1 are directed against conformational epitopes located in the proximity of the glycan at position 332 in V3. Moreover, the NAb activity against all 3 pseudoviruses measured over time showed a notable enhancement (Fig. 5C–E). And the correlation analysis suggested that the neutralization strength against three pseudoviruses were correlated with sera‐specific IgG levels (Fig. S9A–C). These data indicate that improving the B cell immune responses of BLP‐PAM might lead to an increased strength and breadth of NAb response.

In summary, these results suggested that in macaques, IM immunizations with BLP‐PAM were able to induce NAbs against the heterologous tier 1 and tier 2 viruses.

### rAd2‐gp120AE boost enhanced nasal and systemic immune responses

**Fig. 6:**
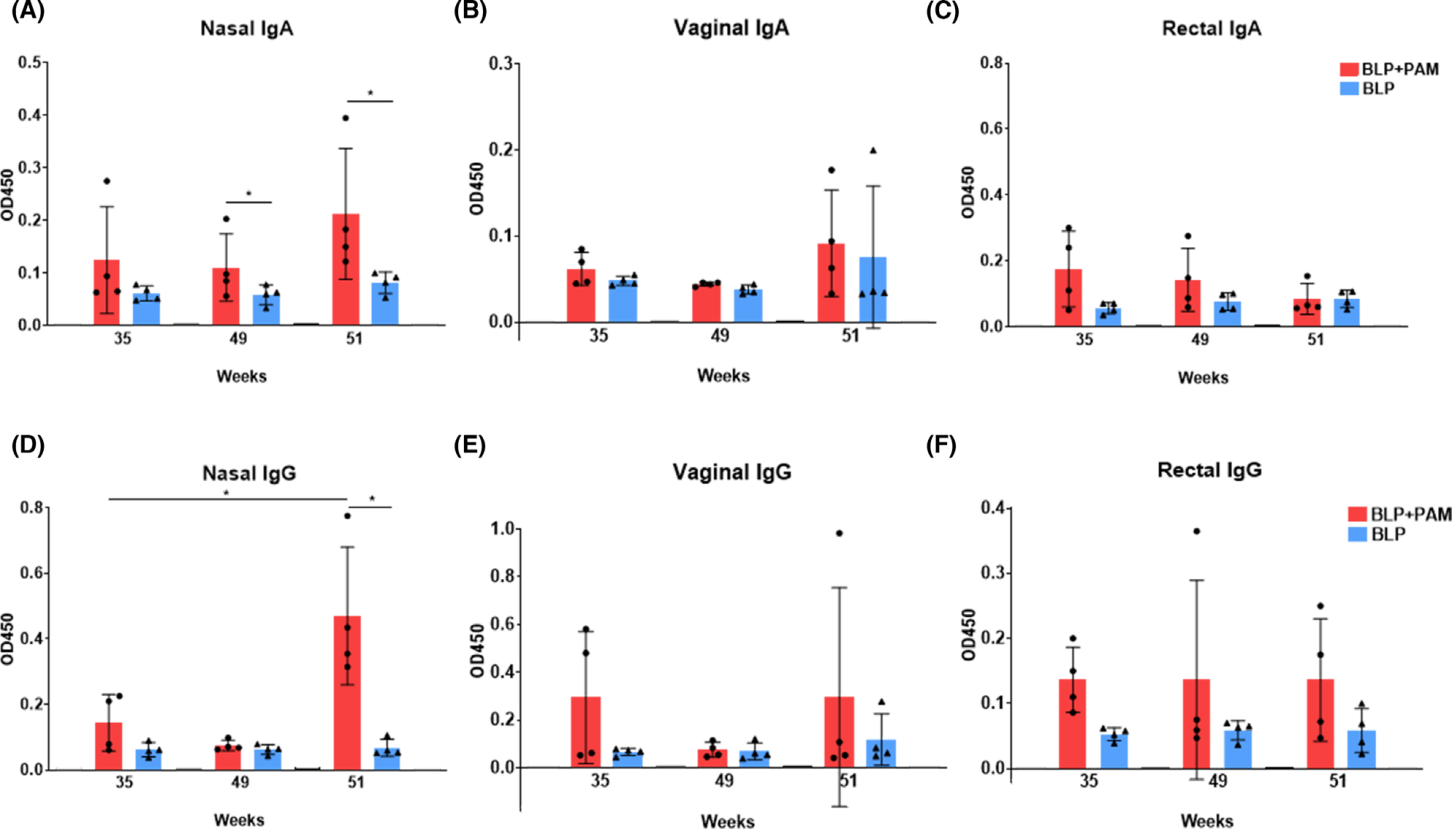
The mucosal immune responses of rhesus macaques boosted with rAd2‐gp120AE. A–C. IgA levels in nasal, rectal and vaginal washings. D–F. IgG levels in nasal, rectal and vaginal washings. Two‐tailed Mann–Whitney *U* test. Mean and standard deviation are indicated with lines and error bars, *P < 0.05.

It has been reported that recombination of the replication‐incompetent adenovirus (rAd2) expressing Zaire ebolavirus glycoprotein induced stronger antibody and cell‐mediated immune responses (Feng *et al*., 2018). Moreover, IN preimmunization with HPV‐16 E7 delivery using live *Lactococcus lactis* in a mouse model significantly enhanced immune response effectiveness to HPV (Rangel‐Colmenero *et al*., 2014). To further improve mucosal and systemic immune responses, the macaques from both groups were boosted with 1 × 10^11^ vp rAd2‐gp120AE (a rAd2 carrying the HIV‐1 gp120 of the AE subtype) via IN and IM routes equally at week 49, 16 weeks after the last IM immunization with BLP‐PAM. At week 51, the IgA and IgG levels, observed in the nasal samples of the animals from the BLP‐PAM group, increased compared with 35 weeks and significantly higher than BLP group; however, there were no significant changes in IgA and IgG levels observed in the rectal and vaginal samples. Nevertheless, almost no IgA and IgG responses were detected in mucosal samples obtained from monkeys from the BLP group, except for one of the macaques, which showed low IgA levels in the vaginal samples following rAd2‐gp120AE immunization (Fig. 6). Additionally, we found that the specific IgG and sIgA levels in rectum washing samples observed at week 49 were maintained following the IM boost (Fig. 6C).

**Fig. 7:**
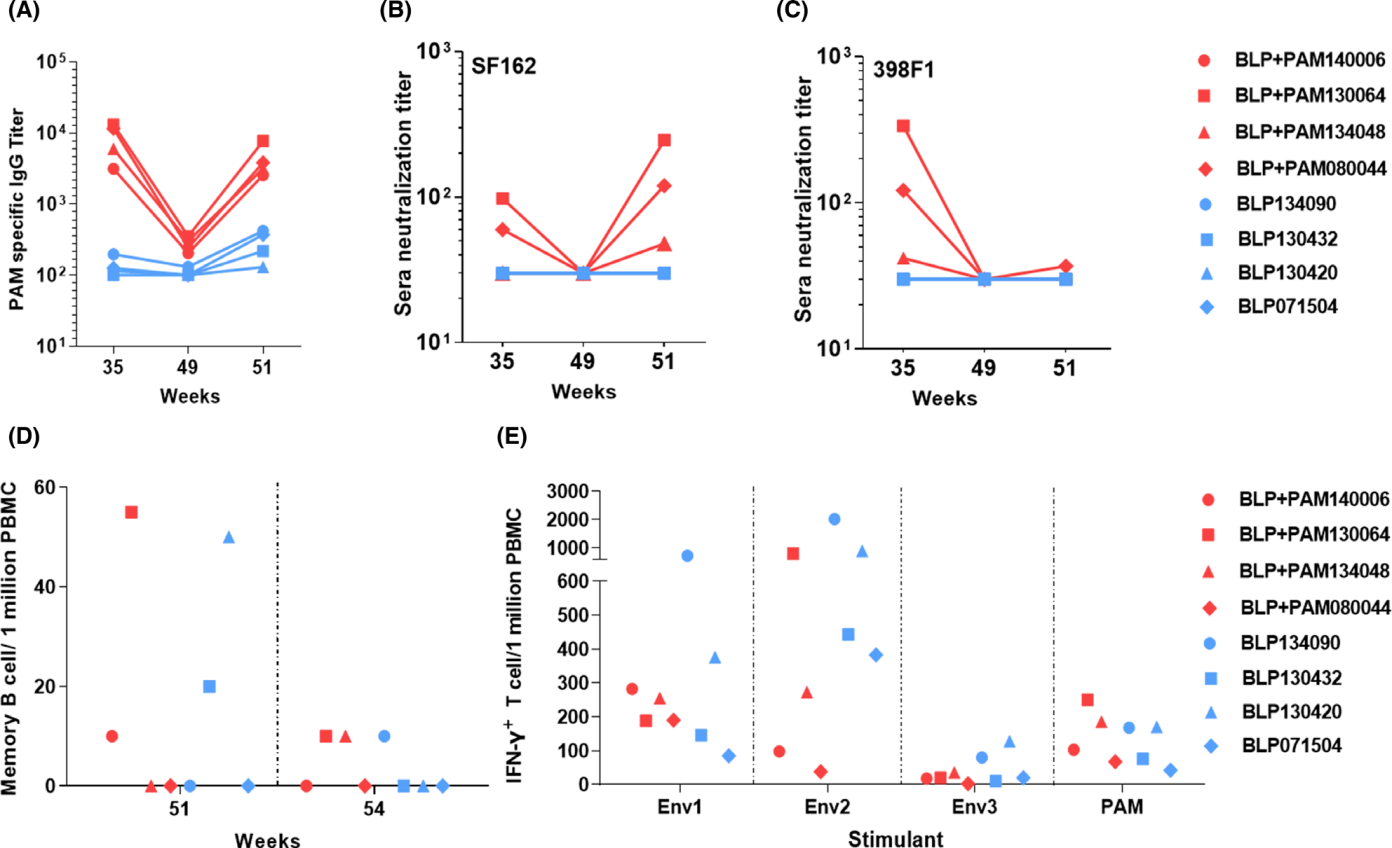
Systemic immune responses after receiving a boost with Ad2‐gp120AE in vaccinated rhesus macaques. A. PAM‐specific IgG end‐point titres at weeks 35, 49 and 51. B–C. Neutralization titres for sera of rhesus macaques tested against SF162 and 398F1. D. PAM‐specific IgG memory B cells among PBMCs measured using a B cell ELISPOT assay at weeks 51 and 54. E. IFN‐γ^+^T cells per million PBMCs, stimulated with Env peptide pools and PAM at week 54.

For the systemic immune responses, HIV‐1 Env‐specific IgG and NAb serum levels were undetectable at week 49, while rebounding after the rAd2‐gp120AE boost in the BLP‐PAM preimmunization group. Furthermore, there were moderate IgG responses in serum samples of the BLP preimmunization animals (Fig. 7A). The correlation plots suggested that the nasal HIV‐1 Env‐specific IgG levels were correlated with sera‐specific IgG levels in the BLP‐PAM preimmunization group at week 51 (Fig. S9D). At week 49, the neutralization activity against tier 1 virus SF162 observed in the BLP‐PAM serum samples was undetectable; however, it was detected again during week 51. Nevertheless, the NAbs against the tier 2 virus 398F1 could not be elicited by the rAd‐gp120AE boost immunization. Alternatively, no NAbs against SF162 or 398F1 were detected in the BLP serum samples (Fig. 7B,C).

To further evaluate the persistence of the PAM‐specific responses generated by rAd2‐gp120AE, HIV‐1 Env‐specific ASCs were quantified 2 and 5 weeks after the rAd2‐gp120AE immunization in order to enumerate antigen‐specific memory B cells. As shown in Fig. 7D, two macaques from each group exhibited a similar number of circulating IgG ASCs after one month, following the immunization with rAd2‐gp120AE. Lastly, T cell responses were analysed using ELISPOT assays. A strong cellular immune response was detected in both groups (week 54), following the stimulation of PBMC with two of the three peptide pools (Fig. 7E).

Collectively, these results showed that the rAd2‐gp120AE boost, via IN and IM routes, further increased the level of nasal IgA and IgG levels and expanded the T cell immune responses in PBMC of BLP‐PAM vaccinated macaques.

## Discussion

**Fig. 8:**
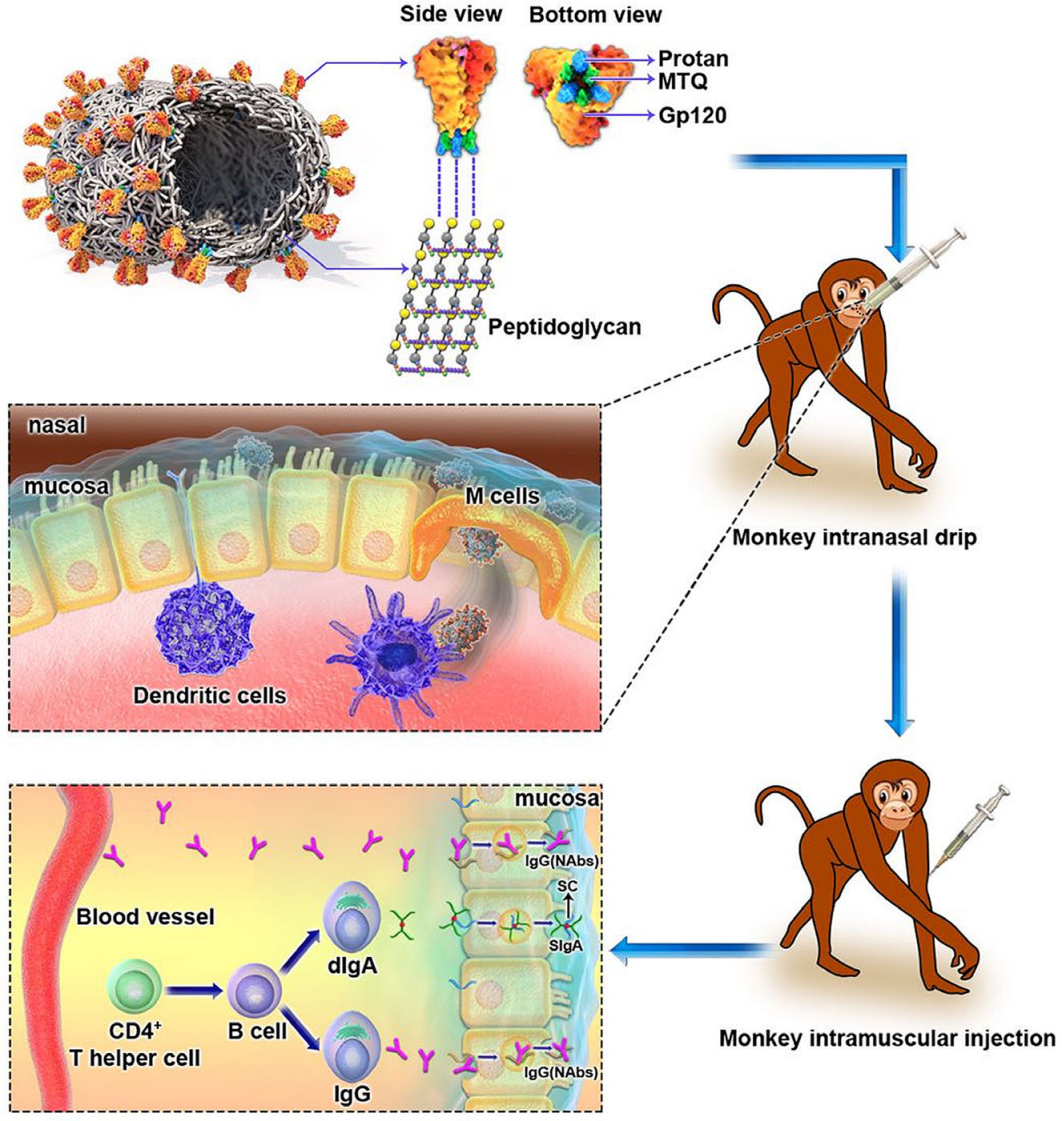
Graphical abstract for immunization strategy and immune responses.

AIDS is mainly a sexually transmitted disease. It is necessary to find an effective anti‐HIV‐1 strategy to impede HIV transmission at the mucosal level (Tudor *et al*., 2009). Eliciting an efficient immune response including sIgA or neutralizing IgG to block the viral adherence to the epithelial cells, prevent transcytosis and neutralize the viruses in epithelial cells is the desirable goal of a mucosal vaccine design (Huang *et al*., 2005; Zhou and Ruprecht, 2014). Preclinical research showed that passively administered bNAbs can protect macaques from the SHIV challenge even with different relative potency (Pegu *et al*., 2014). Therefore, the design of vaccines and immune strategies capable of inducing both mucosal and systemic neutralization responses is expected to provide the ideal protection for healthy individuals. In this study, we first confirmed that a single BLP‐based gp120 trimer vaccine can successfully induce HIV‐1‐specific mucosal responses and serum neutralization antibodies in rhesus macaques via conventional IN and IM immunizations (Fig. 8).

Mucosal immunization with non‐replicative antigens requires the use of adjuvants and delivery systems to break mucosal tolerance and facilitate the uptake of immunogens. Compared with the shortcomings of other delivery systems, lactic acid bacteria, which have consistently been used in food production, are gram‐positive and nonpathogenic; they are considered to have high potential as a delivery system for heterologous proteins that is safe, requires low labour to produce and cost‐effective (Szatraj *et al*., 2017). *Lactobacillus* has been used as a living bacterial adjuvant for an SIV vaccine administered to macaques via vaginal or oral routes (Andrieu *et al*., 2014). Furthermore, engineered *Lactobacillus plantarum* was also used for displaying HIV‐1 Gag using surface anchoring strategies (Kuczkowska *et al*., 2015). In this study, we demonstrated for the first time that the gp120 trimers bound to non‐living particles based on food‐grade non‐recombinant *L. lactis* were able to rapidly induce in situ and distal mucosal immune responses in rhesus macaques following IN immunization, as the sIgA was detectable in nasal, vaginal and rectal washing samples after the first immunization (Fig. 3). In addition, a boost IM immunization using the same vaccine helped increase the IgG levels in mucosal tissues and maintain the mucosal IgG and sIgA levels for more than four months, although it was unable to increase the sIgA levels (Fig. 6). We found that although the vaccine is administered via the nasal route, the strongest mucosal responses were observed in the rectal mucosa. This is beneficial for the HIV‐1 vaccine. Moreover, Musich *et al*. (2015) showed that mucosal IgA from vaccinated and SIV‐infected rhesus macaques is predominant on the gastrointestinal surface and faeces, and they proposed that the faecal matter could be used for IgA collection and purification. We did not collect faecal samples, and thus, did not evaluate the neutralizing activity of mucosal IgA, as we were unable to get enough purified IgA from small volumes of secretory fluids.

Additionally, the BLP‐PAM IM immunization boost without any adjuvants was able to significantly enhance the humoral immune responses in rhesus macaques (Fig. 4). As PAM appears to be a mimic of the native gp120 trimer, as indicated by the antigenic profile analysis, we focused on the NAb responses induced by BLP‐PAM. While the genetic variability of HIV‐1 is among the most challenging problems, studies on the representative vaccines Vax003 and Vax004, which aimed to elicit neutralization antibodies, have provided researchers with the understanding that preclinical antibody neutralization activities should be evaluated using tier 2 and tier 3 pseudoviruses, rather than the TCLA virus (Balasubramanian *et al*., 2018). In our study, vaccine‐induced NAb responses were assessed using tier 1 autologous and standardized 12 global panel of tier 2 pseudoviruses, which were highly sensitive to most bNAbs and exhibited genetic and geographic diversity (deCamp *et al*., 2014). Antibodies binding to the envelope surface spike proteins represent a core machinery that directly neutralizes virions (Burton and Hangartner, 2016). The definition of tier was mainly based on the dynamic nature of Env trimers and the transition time (Montefiori *et al*., 2018). Our neutralization assay results confirmed that BLP‐PAM can elicit neutralizing activity against the heterologous tier 1 and tier 2 viruses following three IM immunizations, although the NAbs titres need to be further improved. The NAb titres increased with antibody binding titres suggests that the intensity and breadth of NAbs may be further enhanced by optimizing the immunization protocol, such as increasing the antigen dose to increase the intensity of the humoral immune responses. Additionally, the optimization of our antigen sequence and structure might assist in enhancement of the vaccine’s ability to induce bNAbs. However, compared with the persistent mucosal responses, serum IgG and NAbs declined to low levels, four months after the last immunization (Fig. 7). This might be explained by the low levels of PAM‐specific IgG memory B cells among PBMCs at week 39 (Fig. 4). Another notable result is that the N332A substitution completely abrogated 398F1 neutralization in all three seropositive monkeys, while the antigenic analysis showed that multiple conformational neutralizing epitopes of PAM can be well exposed. We will carry out more in‐depth analyses to investigate whether this is associated with the BLP carrier.

Although we verified that BLP‐PAM can elicit HIV‐1‐specific mucosal and systemic immune responses through IN and IM immunization combination strategies in rhesus macaques, the overall immune response is relatively weak. T cell responses play an important role in controlling viral replication during natural infection (Dominguez‐Villar *et al*., 2015) and in vaccination settings (Hansen *et al*., 2011). Furthermore, a T cell‐inducing vaccine prevented mucosal SHIV infection even with lower NAb levels (Arunachalam *et al*., 2020). More importantly, recent studies suggested that HIV Env‐specific CD4^+^ T cells might help promote B cell responses in germinal centres (Lee *et al*., 2021; Pusnik *et al*., 2021). As the T cell responses induced by BLP‐PAM were undetectable in our study (Fig. S6), we aimed to compensate for this with a combined viral vector vaccine. Recombinant adenovirus (rAd) vectors are potent inducers of cellular immunity (Feng *et al*., 2020) and have been shown to induce mucosal immunity (Ko *et al*., 2019; Hassan *et al*., 2020). Our results showed that a single injection with rAd2‐gp120AE (via both IN and IM routes) could induce a strong ENV‐specific T cell immune response in rhesus macaques, both in the BLP and BLP‐PAM groups. However, we found that the adenovirus vector vaccine elicited a part of memory B cells induced by BLP‐PAM, while failing to further increase the IgG and NAb levels (Fig. 7). In addition, IN immunization with rAd2‐gp120AE was only capable of improving the immune responses within the nasal mucosa in the BLP‐PAM group, which is consistent with previous studies investigating the Ad5 vector vaccine (Kaufman *et al*., 2010).

In summary, the BLP displaying HIV‐1 gp120 trimer vaccine, in combination with a IN/IM strategy, will help prevent the sexual transmission of HIV by inducing B cell immune responses in the mucosal tissues and blood. In addition, the BLP delivery system provides a potential platform to assist in the development and design of vaccines against other viral pathogens such as influenza or SARS‐CoV‐2.

## Experimental procedures

### Ethics statement

The animals were kept in the Animal Experimental Center of Guangzhou institute of Biomedicine and Health from the Chinese Academy of Sciences, and all experimental procedures were approved by Institutional Animal Care and Use Committee (IACUC:2018034).

### Immunogens and vaccine preparation

The HIV‐1 gp120 trimer PAM were expressed in transient transfected HEK293‐6E suspension cells and extracted from culture through lectin affinity chromatography. Further, the trimeric proteins were purified using size exclusion chromatography (SEC) and analysed using Native PAGE™ 3–12% Bis‐Tris Protein Gels (Invitrogen, Carlsbad, CA, USA). The purity and molecular weight of PAM were examined using HPLC with thyroglobulin (669 kDa), ferritin (440 kDa) and BG505 UFO as control, and the TSK‐GEL G5000PW column (TOSOH, Japan) was used. Vaccine preparation was performed as previously described (Bi *et al*., 2020). Briefly, PAM trimers were mixed with BLPs by shaking overnight at 4°C, centrifuged at 12 000 rpm for five min and resuspended three times in 1× phosphate‐buffered saline (PBS).

### Bio‐layer interferometry

The kinetics analysis of PAM binding to bNAbs PGT121, PG9, 3BNC117 and NAb 447‐52D was performed using Octet Red96 instrument (Forte Bio, Fremont, CA, USA). The samples were added into solid black 96‐well plates (Forte Bio) at 30°C with agitation set to 1000 rpm in 1× PBS [supplemented with 0.1% bovine serum albumin (BSA, Dingguo, Beijing, China) and 0.02% Tween (Dingguo, Beijing, China)]. Final volume for the final solutions was 200 μl per well. The loading process was performed for 300 s, to load 5 μg μl^−1^ of antibody in 1× PBS onto the surface of anti‐human Fc Capture Biosensors (AHC, Forte Bio). The baseline process was performed for 60 s before the association of the antibody from the sensor to the trimer protein in 1× PBS for 300 s. A 6‐step serial titration for the trimer concentration was set with a twofold gradient starting at 800 nM. The dissociation was performed for 300 s in 1× PBS. Double reference subjecting was performed to subtract the background effect of sensor binding to the antibody without the trimer and sensor binding to the trimer without the antibody. Octet data were processed using the Forte Bio’s data acquisition software v.9.0. Experimental data were fitted using a global fit 1:1 model to determine the *K*_D_ values and other kinetic parameters.

### Transmission electron microscopy (TEM)

A total of 10 μg PAM trimer and 5 μl (40 mg ml^−1^) BLP were incubated on a shaking table at 4°C overnight, followed by 5 min centrifugation and resuspension in 1 ml of 1× PBS, three times. BLP (5 μl, 40 mg ml) was obtained following the same protocol as for the control. Further, 10 μl aliquots of BLP‐PAM and BLP were separately applied onto a carbon‐counted 400 Cu mesh grid for 20 min and then blocked for 10 min using 5% BSA in 1× PBS. Next, 10 μg ml^−1^ VRC01 PBS containing 1% BSA was applied on the grid for 90 min, followed by three washing steps with 1× PBST (1 l PBS and 2 ml Tween‐20). Immunogold goat anti‐human IgG conjugates were applied onto the grid for 30 min, followed by washing with 1× PBST three times. Lastly, the samples were negatively stained with uranyl acetate for 90 s, washed for 2 min with pure water and air‐dried. TEM studies were conducted using a transmission electron microscope (FEI Talos L120C, Thermo Fischer, Waltham, MA, USA) operating at 120 keV.

### Analysis of antigenicity using enzyme‐linked immunosorbent assay (ELISA)

ELISA was used to determine the antigenicity and binding capacity of the trimer to BLP. A total of 10 μg PAM trimer and 5 μl (40 mg ml^−1^) BLP were incubated on a shaking table at 37°C for 2 h and then centrifuged, resuspended in PBS and re‐collected; subsequently, the precipitate was dissolved in 10 ml coating buffer (0.05 M carbonate‐bicarbonate, pH 9.6). The final coating buffer was used to coat each well of a 96‐well plate (Jet Biofil, Guangzhou, China). Simultaneously, 10 μg PaC (unrelated Norovirus P particles tagged with Protan protein) and 5 μl (40 mg ml^−1^) BLP were prepared as described for the PAM trimer, while 5 μl (40 mg ml^−1^) BLP was incubated with 10 μg BSA on one plate as negative control. For the positive control, the plate was coated with 10 μg ml^−1^ PAM trimer. ELISA plates were washed three times with 1× PBST using microporous plate washing machine (BioTek, Winooski, VT, USA.). Furthermore, 5% BSA was used to block the plates at 37°C for 2 h. Next, the anti‐HIV‐1 mAbs (VRC01, N6, HJ16, 3BNC117, VRC‐CH31, PG9, PGT145, 697‐30D, CH59, HG120, 2G12, PGT121, PGT126, 10‐1074, PGT128, 447‐52D, F105, 35O22 and 4E10) that were obtained from the NIH AIDS Reagent Program (https://www.aidsreagent.org/) were diluted to a concentration of 1 μg ml^−1^ in 1× PBS supplemented with 1% BSA. Serially diluted mAbs were added and incubated at 37°C for 2 h, followed by three washing steps with 1× PBS. Goat anti‐human IgG horseradish peroxidase (Jackson, West Grove, PA, USA) was added for 1 h (1:5000 in 1% BSA), followed by five washes with 1× PBST. Next, the samples were incubated with TMB (Trans, Beijing, China) staining solution for 6 min, and 2 M H_2_SO_4_ was used to stop the reaction. Lastly, the absorbance was measured at 450 nm using the iMark™ Microplate Reader (Bio‐Rad, Hercules, CA, USA).

### Animal immunization and sample collection

Eight adult female rhesus macaques of Chinese origin aged 6–14 years and weighing 4.8–11.6 kg were assigned to the study (Table S2). The rhesus macaques were randomly divided into two groups: four macaques were assigned to the experimental group immunized with BLP‐PAM (1 mg BLP and 30 μg PAM per macaque each time), and the other four macaques were assigned to the control group immunized with BLP (1 mg BLP per macaque each time). The immunizations were performed at weeks 0, 4, 8, 12 and 22. Furthermore, the immunization dose was doubled at weeks 27 and 33, when IM immunization was performed. All macaques were immunized seven times and the samples were collected two weeks after each immunization. Nasal samples were collected by rinsing the back of the trachea towards the nostrils, and the collected samples were suspended in 1× PBS. Vaginal and rectum samples were collected by washing the cavities using 1× PBS. Blood samples were collected, and peripheral blood mononuclear cells (PBMCs) were isolated as previously described (Sun *et al*., 2013). All macaques were boosted with 1 × 10^11^ vp‐rAd2‐gp120AE (a recombinant replication‐incompetent adenovirus serotype 2 carrying the gp120 of the AE subtype) via IN and IM routes equally.

### Analysis of antibody response in serum

The 96‐well plate was coated with PAM trimer (50 μg per well) overnight at 4°C. After the block using 5% BSA, serum samples diluted 1:100 were added to the plates with a 6‐step serial titration for the serum was set with a fivefold gradient dilution and incubated at 37°C for 2 h. After washing with 1× PBST, goat anti‐monkey IgG (Mabtech, Nacka Strand, Sweden) and IgA (Jackson, West Grove, PA, USA) secondary antibodies, conjugated with HRP, were added and incubated at 37°C for 1 h (1:5000 in 1% BSA). Goat anti‐monkey IgG1 and IgG2 (Mabtech, Sweden) conjugated to biotin (dilution 1:3000) were used as secondary antibodies for the analysis of antibodies subtype and then washing and incubated by Streptavidin‐Alkaline labelled with horseradish peroxidase (dilution 1:1000). The subsequent procedures were the same as the aforementioned description for the antigenicity analysis.

### Analysis of antibody response in mucosal sample

For the IgA and IgG titre analysis, a 96‐well plate was coated with PAM trimer (500 μg per well) overnight at 4°C. After the blocking step, nasal, vaginal and rectal washing samples were centrifuged at 3000 rpm for 5 min, and the supernatants were added to the plates in twofold dilutions and incubated at 37°C for 2 h. The subsequent procedures were the same as the aforementioned description for the serum antibody response analysis, except that the incubation time with TMB was 15 min. For SC analysis goat anti‐monkey SC (Nordic, Susteren, Netherlands) conjugated horseradish peroxidase (dilution 1:1000) was used as secondary antibody.

### Pseudovirus neutralization assay

Neutralization assays were performed as previously described (Bi *et al*., 2020). Briefly, sera of macaques were inactivated and incubated with the global panel of 12 tier 2 and 2 tier 1 HIV‐1 pseudoviruses, which were used to test the neutralization potency and breadth at a 1:3 dilutions at 37°C for 1 h. Next, the mixture was used to infect TZM‐bl cells, and the neutralization was tested after 48 h of incubation. The 398F1 mutants, carrying the N160K, N276D and N332A mutations, were used to test the epitope targeted by serum antibodies. The neutralization titres are reported as ID_50_ titres, and all neutralizing antibody data panels show the geometric mean of titres with geometric SD.

### Cellular immune assay

PAM and peptide pools of Env were used to assess the cellular immune responses as previously described (Sun *et al*., 2013). The Cellular immune assay included IFN‐γ T cell enzyme‐linked immune absorbent spot (ELISPOT) and the multi‐colour intracellular cytokine staining (ICS) for analysing T lymphocyte poly‐functionality.

For IFN‐γ ELISPOT, fresh isolated PBMCs were added in anti‐monkey IFN‐γ monoclonal antibody (BD Pharmingen, San Diego, CA, USA) pre‐coated sterile 96‐well plates containing Immobilon‐P membrane (Millipore, Billerica, MA, USA). PAM or Env peptide pools were added into plates for 24 h stimulation, and then the plates were incubated with a polyclonal anti‐monkey IFN‐γ biotinylated detection antibodie and developed with alkaline phosphatase‐conjugated streptavidin (BD PharMingen, USA) and NBT/BCIP reagent (Pierce, Rockford, lL, USA). Finally, the spots were counted with an ELISPOT reader (Bioreader 4000, Bio‐Sys, Karben, Germany).

For Multi‐colour ICS assay, fresh isolated monkey PBMCs were seeded into 96‐well plates and stimulated with Env peptide pools for 2 h, and then brefeldin A (BD Phamingen, USA) was added and incubated for another 12–16 h. PBMCs were then harvested, stained with LIVE/DEAD fixable dead cell stains to removed dead cells, and then stained with surface antibodies (CD3‐Pacific blue, CD4‐FITC; BD Biosciences, San Jose, CA, USA) for 30 min. Next, the cells were permeabilized and stained with intracellular antibodies (IFN‐γ‐PE, IL‐2‐APC, TNF‐α‐PE‐Cy7; BD Biosciences, USA). The information of these antibodies was summarized in Table S3. Finally, the cells were detected with an LSR Fortessa instrument (BD Biosciences).

### Memory B cell ELISPOT assay

PBMCs were pre‐activated with TLR agonist R848 (1 μg ml^−1^, InvivoGen) and human IL‐2 (100 unit per ml, PeproTech) for 3 days. The activated B cells were washed and performed ELISPOT assay as previously described (Wu *et al*., 2018). Briefly, MultiScreen 96‐well plates were coated with PAM trimer (1 μg per well) or anti‐monkey IgG antibodies (1 μg per well). 2 × 10^5^ cells were seeded for the detection of PAM trimer‐specific IgG‐secreting cells, or 5 × 10^4^ cells for the detection of total IgG‐secreting cells. After overnight incubation, cells were lysed and probed with HRP‐conjugated rabbit anti‐monkey IgG (H&L). Spots were developed using 3‐amino‐9‐ethylcarbazole (AEC) substrate (BD Pharmigen). Spots of antibody‐secreting cells (ASC) were counted using an ELISPOT reader and data are presented as the number of memory B cells per million cells.

### Statistical analysis

Statistical analysis was conducted using GraphPad Prism (version 8.0; La Jolla, CA, USA) and Microsoft Excel. *P*‐values < 0.05 were considered to be statistically significant (indicated with an asterisk, *). The correlation analysis was determined by non‐parametric Spearman and Mann–Whitney tests.

### Data analysis

The authors declare that all relevant data are available from the corresponding author upon request.

## Conflict of interest

The authors declare that they have no conflict of interest.

## Author contributions

H.Y.W., J.P.B., B.Y., L.C. and X.H.Y. contributed to conceptualization. H.Y.W., P.C.L, M.Z., J.P.B., Y.Z.H., F.S.L., F.G. and W.K. contributed to methodology. H.Y.W., P.C.L, M.Z., J.P.B., Y.Z.H. F.S.L. and R.Z.Y contributed to investigation. H.Y.W. contributed to writing – original draft. H.Y.W., P.C.L, Y.Z.H., B.Y., L.C. and X.H.Y. contributed to writing – review and editing. H.Y.W. contributed to visualization. B.Y., L.C. and X.H.Y. contributed to supervision and project administration. B.Y., L.C. and X.H.Y. contributed to funding acquisition.

## Supporting information


**Fig. S1**. SEC profile of High Molecular Weight Calibration Kit and lectin‐purified BG505 UFO expressed in 293‐6E cells. A TSK‐GEL G5000PW column was used. A. Ferritin, 669 kDa. B. Catalase, 440 kDa. C. BG505 UFO.
**Fig. S2**. Transmission electron microscopic images of single BLP‐PAM and BLP. Typical examples are shown. A. BLP‐PAM coated with VRC01 and then immunogold labelling of goat‐anti‐human IgG. B. ‘Empty’ BLP coated with VRC01 and immunogold labelling of goat‐anti‐human IgG.
**Fig. S3**. Antigenicity analysis of PAM and BLP‐PAM by ELISA. A–C. Representative epitope exposing analysis of CD4bs (non‐NAb of F105). D–F. Representative binding curves of quaternary structure dependent bNAbs PG9, PG16, PGT145 targeting V1V2 Apex. G–I V3‐glycan (10‐1074), 447‐52D (V3‐loop), gp120 V2 (CH59). Note that the scales on the *y*‐axes and *x*‐axes vary from mAb to mAb. BLP and BLP‐PaC were set as control.
**Fig. S4**. The mucosal immune responses in vaccinated rhesus macaques. A, B. Secretory component (SC) levels in nasal and vaginal washings between week −2 and week 35 measured by ELISA. Mean and standard deviation are indicated with lines and error bars.
**Fig. S5**. Isotype analysis of PAM specific antibody in sera. A–D. IgG isotype analysis in sera two weeks after pre‐immunization and intramuscular immunization as measured by ELISA. E. Ratio of IgG1/IgG2 in sera.
**Fig. S6**. T cell immune responses in vaccinated rhesus macaques. A. IFN‐γ^+^ secreting T cells per million PBMCs stimulated by Env peptide pools and “empty” BLP at 39 weeks PBMC measured by ELISPOT. B–D. The percentages of IL‐2^+^, TNF‐α^+^ and IFN‐γ^+^ CD4^+^ T cells in PBMC measured by FACS. PMA and ionomycin were used as control.
**Fig. S7**. Neutralization titers (ID50) for sera from rhesus macaques tested against a panel of Env‐pseudotyped viruses and mutants. A. Neutralization breath on Tier1 and Tier2 panel from BLP‐PAM group sera samples. B. Neutralization breath on Tier 1 and Tier 2 panel from BLP group serum samples.
**Fig. S8**. Neutralization titers (ID50) from serum samples of rhesus macaques in BLP‐PAM group tested against 398F1 mutants.
**Fig. S9**. Correlation analysis for antibody responses. A–C. Correlation between binding antibody responses of sera to PAM and NAbs to Tier‐1 (SF162 and MW965) Tier‐2 viruses 398F1at week 35. C. Correlation between the IgG levels of sera and nasal washings at week 51. The Pearson correlation coefficient, *r*, calculated using spss software version 22.0 for the respective correlations are given.
**Fig. S10**. The humoral and mucosal responses of BLP‐PAM in rhesus macaques. A. PAM specific IgG antibodies binding endpoint titers in sera measured by ELISA. B. PAM specific sIgA antibodies level in rectal samples.
**Table S1.** Kinetic analysis of Protan‐gp120AE‐MTQ and BG505 UFO binding to bNAbs and non‐bNAbs by Bio‐layer interferometry. Table shows Kon, Koff, KD.
**Table S2.** Information of Chinese rhesus macaques used in this study.
**Table S3.** Antibodies used for analytical flow cytometry in this study.

## References

[mbt214022-bib-0001] Allers, K. , Puyskens, A. , Epple, H.‐J. , Schürmann, D. , Hofmann, J. , Moos, V. , and Schneider, T. (2016) The effect of timing of antiretroviral therapy on CD4+ T‐cell reconstitution in the intestine of HIV‐infected patients. Mucosal Immunol 9: 265–274. 26129649 10.1038/mi.2015.58

[mbt214022-bib-0002] Andrabi, R. , Bhiman, J.N. , and Burton, D.R. (2018) Strategies for a multi‐stage neutralizing antibody‐based HIV vaccine. Curr Opin Immunol 53: 143–151. 29775847 10.1016/j.coi.2018.04.025 PMC6141341

[mbt214022-bib-0003] Andrieu, J.M. , Chen, S. , Lai, C. , Guo, W. , and Lu, W. (2014) Mucosal SIV vaccines comprising inactivated virus particles and bacterial adjuvants induce CD8(+) T‐regulatory cells that suppress SIV‐Positive CD4(+) T‐cell activation and prevent SIV infection in the Macaque Model. Front Immunol 5: 297. 25071760 10.3389/fimmu.2014.00297 PMC4074992

[mbt214022-bib-0004] Arunachalam, P.S. , Charles, T.P. , Joag, V. , Bollimpelli, V.S. , Scott, M.K.D. , Wimmers, F. , *et al*. (2020) T cell‐inducing vaccine durably prevents mucosal SHIV infection even with lower neutralizing antibody titers. Nat Med 26: 932–940. 32393800 10.1038/s41591-020-0858-8 PMC7303014

[mbt214022-bib-0005] Balasubramanian, P. , Williams, C. , Shapiro, M.B. , Sinangil, F. , Higgins, K. , Nádas, A. , *et al*. (2018) Functional antibody response against V1V2 and V3 of HIV gp120 in the VAX003 and VAX004 vaccine trials. Sci Rep 8: 542. 29323175 10.1038/s41598-017-18863-0 PMC5765017

[mbt214022-bib-0007] Benjelloun, F. , Dawood, R. , Urcuqui‐Inchima, S. , Chanut, B. , Verrier, B. , Lucht, F. , *et al*. (2013) Secretory IgA specific for MPER can protect from HIV‐1 infection in vitro. AIDS 27: 1992–1995. 23696073 10.1097/QAD.0b013e3283632ea1

[mbt214022-bib-0008] Bi, J. , Li, F. , Zhang, M.O. , Wang, H. , Lu, J. , Zhang, Y. , *et al*. (2020) An HIV‐1 vaccine based on bacterium‐like particles elicits Env‐specific mucosal immune responses. Immunol Lett 222: 29–39. 32173375 10.1016/j.imlet.2020.03.002

[mbt214022-bib-0009] Burton, D.R. , and Hangartner, L. (2016) Broadly neutralizing antibodies to HIV and their role in vaccine design. Annu Rev Immunol 34: 635–659. 27168247 10.1146/annurev-immunol-041015-055515 PMC6034635

[mbt214022-bib-0010] deCamp, A. , Hraber, P. , Bailer, R.T. , Seaman, M.S. , Ochsenbauer, C. , Kappes, J. , *et al*. (2014) Global panel of HIV‐1 Env reference strains for standardized assessments of vaccine‐elicited neutralizing antibodies. J Virol 88(5): 2489–2507. 24352443 10.1128/JVI.02853-13 PMC3958090

[mbt214022-bib-0011] Demberg, T. , and Robert‐Guroff, M. (2009) Mucosal immunity and protection against HIV/SIV infection: strategies and challenges for vaccine design. Int Rev Immunol 28: 20–48. 19241252 10.1080/08830180802684331 PMC3466469

[mbt214022-bib-0012] Devito, C. , Broliden, K. , Kaul, R. , Svensson, L. , Johansen, K. , Kiama, P. , *et al*. (2000) Mucosal and plasma IgA from HIV‐1‐exposed uninfected individuals inhibit HIV‐1 transcytosis across human epithelial cells. J Immunol 165: 5170–5176. 11046049 10.4049/jimmunol.165.9.5170

[mbt214022-bib-0013] Dominguez‐Villar, M. , Gautron, A.S. , de Marcken, M. , Keller, M.J. , and Hafler, D.A. (2015) TLR7 induces anergy in human CD4(+) T cells. Nat Immunol 16: 118–128. 25401424 10.1038/ni.3036 PMC4413902

[mbt214022-bib-0014] Dubrovskaya, V. , Tran, K. , Ozorowski, G. , Guenaga, J. , Wilson, R. , Bale, S. , *et al*. (2019). Vaccination with glycan‐modified HIV NFL envelope trimer‐liposomes elicits broadly neutralizing antibodies to multiple sites of vulnerability. Immunity 51: 915–929.e7. 31732167 10.1016/j.immuni.2019.10.008 PMC6891888

[mbt214022-bib-0015] Feng, L. , Wang, Q. , Shan, C. , Yang, C. , Feng, Y. , Wu, J. , *et al*. (2020) An adenovirus‐vectored COVID‐19 vaccine confers protection from SARS‐COV‐2 challenge in rhesus macaques. Nat Commun 11(1): 4207. 32826924 10.1038/s41467-020-18077-5 PMC7442803

[mbt214022-bib-0016] Feng, Y. , Li, C. , Hu, P. , Wang, Q. , Zheng, X. , Zhao, Y. , *et al*. (2018) An adenovirus serotype 2‐vectored ebolavirus vaccine generates robust antibody and cell‐mediated immune responses in mice and rhesus macaques. Emerg Microbes Infect 7: 101. 29872043 10.1038/s41426-018-0102-5 PMC5988821

[mbt214022-bib-0017] Gorny, M.K. , Moore, J.P. , Conley, A.J. , Karwowska, S. , Sodroski, J. , Williams, C. , *et al*. (1994) Human anti‐V2 monoclonal antibody that neutralizes primary but not laboratory isolates of human immunodeficiency virus type 1. J Virol 68: 8312–8320. 7525987 10.1128/jvi.68.12.8312-8320.1994 PMC237300

[mbt214022-bib-0018] Hansen, S.G. , Ford, J.C. , Lewis, M.S. , Ventura, A.B. , Hughes, C.M. , Coyne‐Johnson, L. , *et al*. (2011) Profound early control of highly pathogenic SIV by an effector memory T‐cell vaccine. Nature 473: 523–527. 21562493 10.1038/nature10003 PMC3102768

[mbt214022-bib-0019] Hassan, A.O. , Kafai, N.M. , Dmitriev, I.P. , Fox, J.M. , Smith, B.K. , Harvey, I.B. , *et al*. (2020) A single‐dose intranasal ChAd vaccine protects upper and lower respiratory tracts against SARS‐CoV‐2. Cell 183: 169–184.e13. 32931734 10.1016/j.cell.2020.08.026 PMC7437481

[mbt214022-bib-0020] Huang, Y.T. , Wright, A. , Gao, X. , Kulick, L. , Yan, H. , and Lamm, M.E. (2005). Intraepithelial cell neutralization of HIV‐1 replication by IgA. J Immunol 174: 4828–4835. 15814709 10.4049/jimmunol.174.8.4828

[mbt214022-bib-0021] Jones, A.T. , Shen, X. , Walter, K.L. , LaBranche, C.C. , Wyatt, L.S. , Tomaras, G.D. , *et al*. (2019) HIV‐1 vaccination by needle‐free oral injection induces strong mucosal immunity and protects against SHIV challenge. Nat Commun 10(1): 798. 30778066 10.1038/s41467-019-08739-4 PMC6379385

[mbt214022-bib-0022] Julien, J.‐P. , Lee, J.H. , Cupo, A. , Murin, C.D. , Derking, R. , Hoffenberg, S. , *et al*. (2013) Asymmetric recognition of the HIV‐1 trimer by broadly neutralizing antibody PG9. Proc Natl Acad Sci USA 110: 4351–4356. 23426631 10.1073/pnas.1217537110 PMC3600498

[mbt214022-bib-0023] Kang, Z.H. , Bricault, C.A. , Borducchi, E.N. , Stephenson, K.E. , Seaman, M.S. , Pau, M. , *et al*. (2018) Similar epitope specificities of IgG and IgA antibodies elicited by Ad26 vector prime, Env protein boost immunizations in Rhesus monkeys. J Virol 92: 18. 10.1128/JVI.00537-18 PMC6052297 29793950

[mbt214022-bib-0024] Kaufman, D.R. , Bivas‐Benita, M. , Simmons, N.L. , Miller, D. , and Barouch, D.H. (2010) Route of adenovirus‐based HIV‐1 vaccine delivery impacts the phenotype and trafficking of vaccine‐elicited CD8+ T lymphocytes. J Virol 84: 5986–5996. 20357087 10.1128/JVI.02563-09 PMC2876628

[mbt214022-bib-0025] Ko, E.J. , Helmold Hait, S. , Enyindah‐Asonye, G. , Rahman, M.A. , Hoang, T. , and Robert‐Guroff, M. (2019) Replicating adenovirus‐SIV immunization of rhesus macaques induces mucosal dendritic cell activation and function leading to rectal immune responses. Front Immunol 10: 779. 31031768 10.3389/fimmu.2019.00779 PMC6473464

[mbt214022-bib-0026] Kuczkowska, K. , Mathiesen, G. , Eijsink, V.G. , and Oynebraten, I. (2015) Lactobacillus plantarum displaying CCL3 chemokine in fusion with HIV‐1 Gag derived antigen causes increased recruitment of T cells. Microb Cell Fact 14: 169. 26494531 10.1186/s12934-015-0360-z PMC4618854

[mbt214022-bib-0027] Lee, J.H. , Hu, J.K. , Georgeson, E. , Nakao, C. , Groschel, B. , Dileepan, T. , *et al*. (2021) Modulating the quantity of HIV Env‐specific CD4 T cell help promotes rare B cell responses in germinal centers. J Exp Med 218: 1254. 10.1084/jem.20201254 PMC7769167 33355623

[mbt214022-bib-0028] Lim, S.G. , Condez, A. , Lee, C.A. , Johnson, M.A. , Elia, C. , and Poulter, L.W. (1993) Loss of mucosal CD4 lymphocytes is an early feature of HIV infection. Clin Exp Immunol 92: 448–454. 8099858 10.1111/j.1365-2249.1993.tb03419.x PMC1554790

[mbt214022-bib-0029] Montefiori, D.C. , Roederer, M. , Morris, L. , and Seaman, M.S. (2018) Neutralization tiers of HIV‐1. Curr Opin HIV AIDS 13: 128–136. 29266013 10.1097/COH.0000000000000442 PMC5802254

[mbt214022-bib-0030] Morgane, B. , Daniela, T. , Anne‐Sophie, D. , Annette, A. , Yonatan, G. , Marie‐Gaëlle, R. , *et al*. (2011) Neutralization tiers of HIV‐1. Immunization with HIV‐1 gp41 subunit virosomes induces mucosal antibodies protecting nonhuman primates against vaginal SHIV challenges. Immunity 34: 146–148. 21315623 10.1016/j.immuni.2011.01.015

[mbt214022-bib-0031] Mouquet, H. , Scharf, L. , Euler, Z. , Liu, Y. , Eden, C. , Scheid, J.F. , *et al*. (2012) Complex‐type N‐glycan recognition by potent broadly neutralizing HIV antibodies. Proc Natl Acad Sci USA 109: E3268–E3277. 23115339 10.1073/pnas.1217207109 PMC3511153

[mbt214022-bib-0032] Moyer, T.J. , Kato, Y.U. , Abraham, W. , Chang, J.Y.H. , Kulp, D.W. , Watson, N. , *et al*. (2020) Engineered immunogen binding to alum adjuvant enhances humoral immunity. Nat Med 26: 430–440. 32066977 10.1038/s41591-020-0753-3 PMC7069805

[mbt214022-bib-0033] Musich, T. , Demberg, T. , Morgan, I.L. , Estes, J.D. , Franchini, G. , and Robert‐Guroff, M. (2015) Purification and functional characterization of mucosal IgA from vaccinated and SIV‐infected rhesus macaques. Clin Immunol 158: 127–139. 25840105 10.1016/j.clim.2015.03.020 PMC4464970

[mbt214022-bib-0034] Pavot, V. , Rochereau, N. , Lawrence, P. , Girard, M.P. , Genin, C. , Verrier, B. , and Paul, S. (2014) Recent progress in HIV vaccines inducing mucosal immune responses. AIDS 28: 1701–1718. 25009956 10.1097/QAD.0000000000000308

[mbt214022-bib-0035] Pegu, A. , Hessell, A.J. , Mascola, J.R. , and Haigwood, N.L. (2017) Use of broadly neutralizing antibodies for HIV‐1 prevention. Immunol Rev 275: 296–312. 28133803 10.1111/imr.12511 PMC5314445

[mbt214022-bib-0036] Pegu, A. , Yang, Z.‐Y. , Boyington, J.C. , Wu, L. , Ko, S.‐Y. , Schmidt, S.D. , *et al*. (2014) Neutralizing antibodies to HIV‐1 envelope protect more effectively in vivo than those to the CD4 receptor. Sci Transl Med 6: 243–288. 10.1126/scitranslmed.3008992 PMC4562469 24990883

[mbt214022-bib-0037] Pusnik, J. , Fischinger, S. , Dittmer, U. , Esser, S. , van Gils, M.J. , Sanders, R.W. , *et al*. (2021) Production of HIV‐1 Env‐specific antibodies mediating innate immune functions depends on cognate IL‐21‐ secreting CD4+ T cells. J Virol, 95, e02097‐20. 10.1128/JVI.02097-20 PMC8103692 33504598

[mbt214022-bib-0038] Rangel‐Colmenero, B.R. , Gomez‐Gutierrez, J.G. , Villatoro‐Hernandez, J. , Zavala‐Flores, L.M. , Quistian‐Martinez, D. , Rojas‐Martinez, A. , *et al*. (2014) Enhancement of Ad‐CRT/E7‐mediated antitumor effect by preimmunization with *L. lactis* expressing HPV‐16 E7. Viral Immunol 27: 463–467. 25216057 10.1089/vim.2014.0055

[mbt214022-bib-0039] Ruprecht, R.M. , Marasini, B. , and Thippeshappa, R. (2019) Mucosal antibodies: defending epithelial barriers against HIV‐1 invasion. Vaccines 7: 194. 10.3390/vaccines7040194 PMC6963197 31771162

[mbt214022-bib-0040] Sliepen, K. , Han, B.W. , Bontjer, I. , Mooij, P. , Garces, F. , Behrens, A.‐J. , *et al*. (2019) Structure and immunogenicity of a stabilized HIV‐1 envelope trimer based on a group‐M consensus sequence. Nat Commun 10: 2355. 31142746 10.1038/s41467-019-10262-5 PMC6541627

[mbt214022-bib-0041] Steichen, J.M. , Lin, Y.‐C. , Havenar‐Daughton, C. , Pecetta, S. , Ozorowski, G. , Willis, J.R. , *et al*. (2019) A generalized HIV vaccine design strategy for priming of broadly neutralizing antibody responses. Science 366: eaax4380. 31672916 10.1126/science.aax4380 PMC7092357

[mbt214022-bib-0042] Sun, C. , Chen, Z. , Tang, X. , Zhang, Y. , Feng, L. , Du, Y. , *et al*. (2013) Mucosal priming with a replicating‐vaccinia virus‐based vaccine elicits protective immunity to simian immunodeficiency virus challenge in rhesus monkeys. J Virol 87: 5669–5677. 23487457 10.1128/JVI.03247-12 PMC3648167

[mbt214022-bib-0043] Szatraj, K. , Szczepankowska, A.K. , and Chmielewska‐Jeznach, M. (2017) Lactic acid bacteria ‐ promising vaccine vectors: possibilities, limitations, doubts. J Appl Microbiol 123: 325–339. 28295939 10.1111/jam.13446 PMC7166332

[mbt214022-bib-0044] Taha Hirboda, M. , Kaul, R. , Reichard, C. , Kimani, J. , Ngugi, E. , Bwayo, J.J. , *et al*. (2018) HIV‐neutralizing immunoglobulin A and HIV‐specific proliferation are independently associated with reduced HIV acquisition in Kenyan sex workers. AIDS 22: 727–735. 10.1097/QAD.0b013e3282f56b64 18356602

[mbt214022-bib-0045] Tudor, D. , Derrien, M. , Diomede, L. , Drillet, A.‐S. , Houimel, M. , Moog, C. , *et al*. (2009) HIV‐1 gp41‐specific monoclonal mucosal IgAs derived from highly exposed but IgG‐seronegative individuals block HIV‐1 epithelial transcytosis and neutralize CD4(+) cell infection: an IgA gene and functional analysis. Mucosal Immunol 2: 412–426. 19587640 10.1038/mi.2009.89

[mbt214022-bib-0046] Van Braeckel‐Budimir, N. , Haijema, B.J. , and Leenhouts, K. (2013) Bacterium‐like particles for efficient immune stimulation of existing vaccines and new subunit vaccines in mucosal applications. Front Immunol 4: 282. 24062748 10.3389/fimmu.2013.00282 PMC3775300

[mbt214022-bib-0047] Walker, L.M. , Huber, M. , Doores, K.J. , Falkowska, E. , Pejchal, R. , Julien, J.P. , *et al*. (2011) Broad neutralization coverage of HIV by multiple highly potent antibodies. Nature 477(7365): 466–470. 21849977 10.1038/nature10373 PMC3393110

[mbt214022-bib-0048] Walker, L.M. , Phogat, S.K. , Chan‐Hui, P.Y. , Wagner, D. , Phung, P. , Goss, J.L. , *et al*. (2009) Broad and potent neutralizing antibodies from an African donor reveal a new HIV‐1 vaccine target. Science 326: 285–289. 19729618 10.1126/science.1178746 PMC3335270

[mbt214022-bib-0049] Ward, A.B. , and Wilson, I.A. (2015) Insights into the trimeric HIV‐1 envelope glycoprotein structure. Trends Biochem Sci 40: 101–107. 25600289 10.1016/j.tibs.2014.12.006 PMC4310573

[mbt214022-bib-0050] Wright, A. , Lamm, M.E. , and Huang, Y.T. (2008) Excretion of human immunodeficiency virus type 1 through polarized epithelium by immunoglobulin A. J Virol 82: 11526–11535. 18829757 10.1128/JVI.01111-08 PMC2583660

[mbt214022-bib-0051] Wu, T. , Ma, F. , Ma, X. , Jia, W. , Pan, E. , Cheng, G. , *et al*. (2018) Regulating innate and adaptive immunity for controlling SIV infection by 25‐hydroxycholesterol. Front Immunol 9: 2686. 30524435 10.3389/fimmu.2018.02686 PMC6262225

[mbt214022-bib-0052] Zhou, M. , and Ruprecht, R.M. (2014) Are anti‐HIV IgAs good guys or bad guys? Retrovirology 11: 109. 25499540 10.1186/s12977-014-0109-5 PMC4297362

[mbt214022-bib-0053] Zhou, T. , and Xu, K. (2018) Structural features of broadly neutralizing antibodies and rational design of vaccine. Adv Exp Med Biol 1075: 73–95. 30030790 10.1007/978-981-13-0484-2_4

