# Supplementary material for: Vaccine with bacterium‐like particles displaying HIV‐1 gp120 trimer elicits specific mucosal responses and neutralizing antibodies in rhesus macaques
Source: Microb Biotechnol. 2022 Mar 15;15(7):2022–39. doi: 10.1111/1751-7915.14022 (PMC9249329; doi:10.1111/1751-7915.14022)
Supplement: Supplementary file 1 — Fig. S1. SEC profile of High Molecular Weight Calibration Kit and lectin‐purified BG505 UFO expressed in 293‐6E cells. A TSK‐GEL G5000PW column was used. A. Ferritin, 669 kDa. B. Catalase, 440 kDa. C. BG505 UFO. Fig. S2. Transmission electron microscopic images of single BLP‐PAM and BLP. Typical examples are shown. A. BLP‐PAM coated with VRC01 and then immunogold labelling of goat‐anti‐human IgG. B. ‘Empty’ BLP coated with VRC01 and immunogold labelling of goat‐anti‐human IgG. Fig. S3. Antigenicity analysis of PAM and BLP‐PAM by ELISA. A–C. Representative epitope exposing analysis of CD4bs (non‐NAb of F105). D–F. Representative binding curves of quaternary structure dependent bNAbs PG9, PG16, PGT145 targeting V1V2 Apex. G–I V3‐glycan (10‐1074), 447‐52D (V3‐loop), gp120 V2 (CH59). Note that the scales on the y‐axes and x‐axes vary from mAb to mAb. BLP and BLP‐PaC were set as control. Fig. S4. The mucosal immune responses in vaccinated rhesus macaques. A, B. Secretory component (SC) levels in nasal and vaginal washings between week −2 and week 35 measured by ELISA. Mean and standard deviation are indicated with lines and error bars. Fig. S5. Isotype analysis of PAM specific antibody in sera. A–D. IgG isotype analysis in sera two weeks after pre‐immunization and intramuscular immunization as measured by ELISA. E. Ratio of IgG1/IgG2 in sera. Fig. S6. T cell immune responses in vaccinated rhesus macaques. A. IFN‐γ+ secreting T cells per million PBMCs stimulated by Env peptide pools and “empty” BLP at 39 weeks PBMC measured by ELISPOT. B–D. The percentages of IL‐2+, TNF‐α+ and IFN‐γ+ CD4+ T cells in PBMC measured by FACS. PMA and ionomycin were used as control. Fig. S7. Neutralization titers (ID50) for sera from rhesus macaques tested against a panel of Env‐pseudotyped viruses and mutants. A. Neutralization breath on Tier1 and Tier2 panel from BLP‐PAM group sera samples. B. Neutralization breath on Tier 1 and Tier 2 panel from BLP group serum samples. Fig. S8. Neutralizat [file MBT2-15-2022-s001.docx]

Supplementary Materials for

**Vaccine with bacterium-like particles displaying HIV-1 gp120 trimer elicits specific mucosal responses and neutralizing antibodies in rhesus macaques**

**Supplementary Figures**


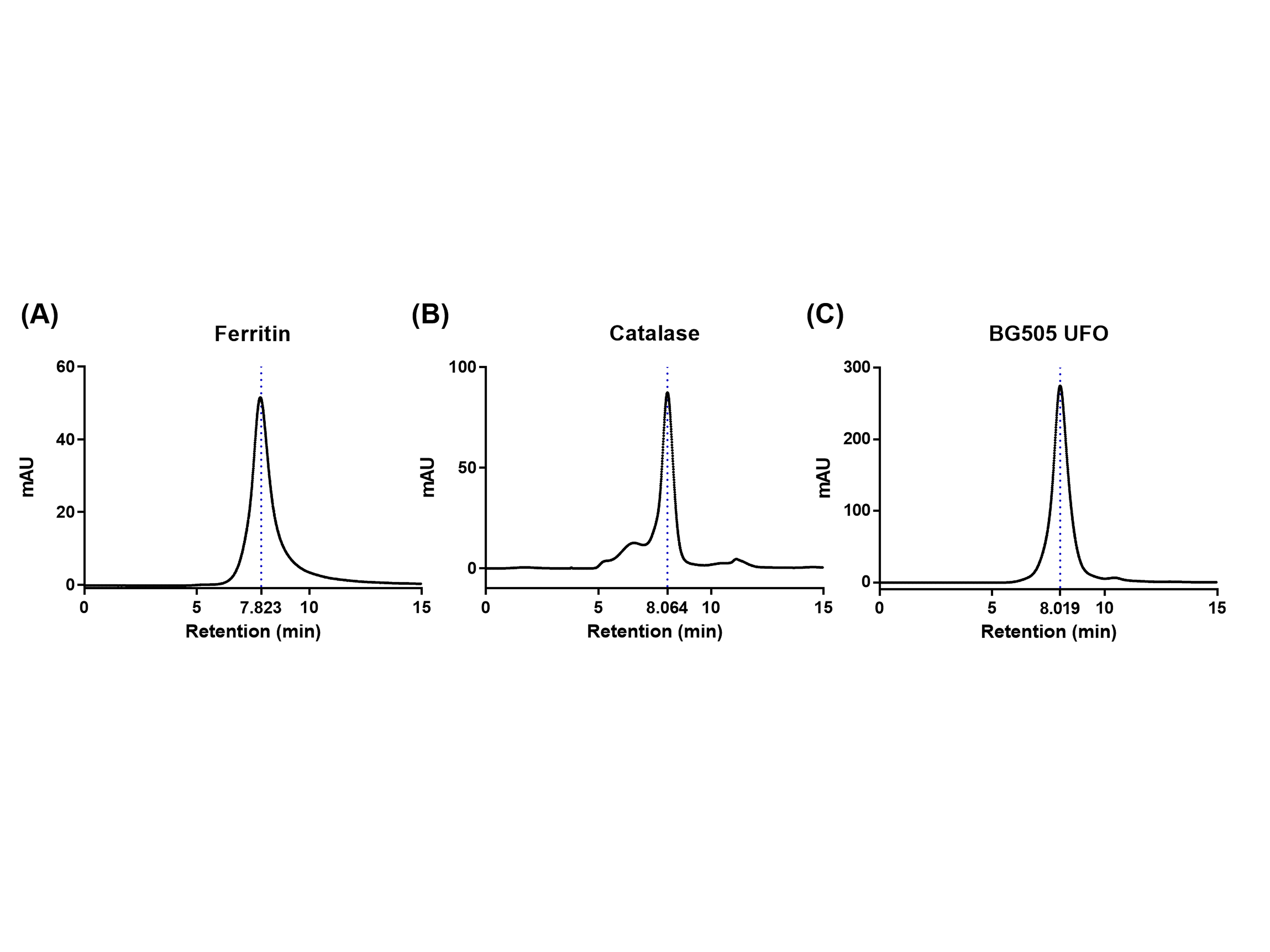


**Fig. S1.** SEC profile of High Molecular Weight Calibration Kit and lectin-purified BG505 UFO expressed in 293-6E cells. A TSK-GEL G5000PW column was used.

1. Ferritin, 669kDa. B. Catalase, 440kDa. C. BG505 UFO.


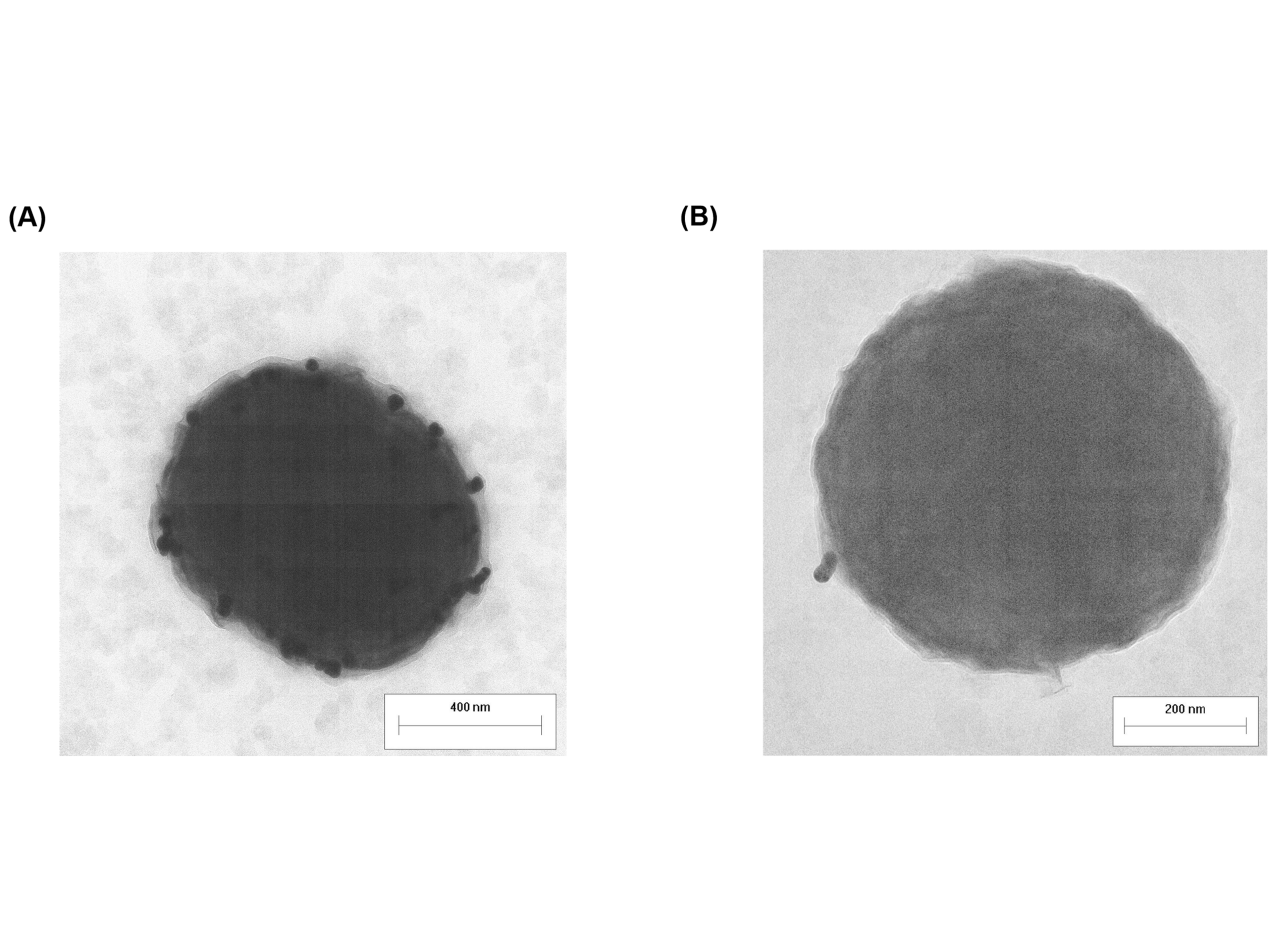


**Fig. S2.** Transmission electron microscopic images of single BLP-PAM and BLP. Typical examples are shown.

1. BLP-PAM coated with VRC01 and then immunogold labelling of goat-anti-human IgG.
2. ‘Empty’ BLP coated with VRC01 and immunogold labelling of goat-anti-human IgG.


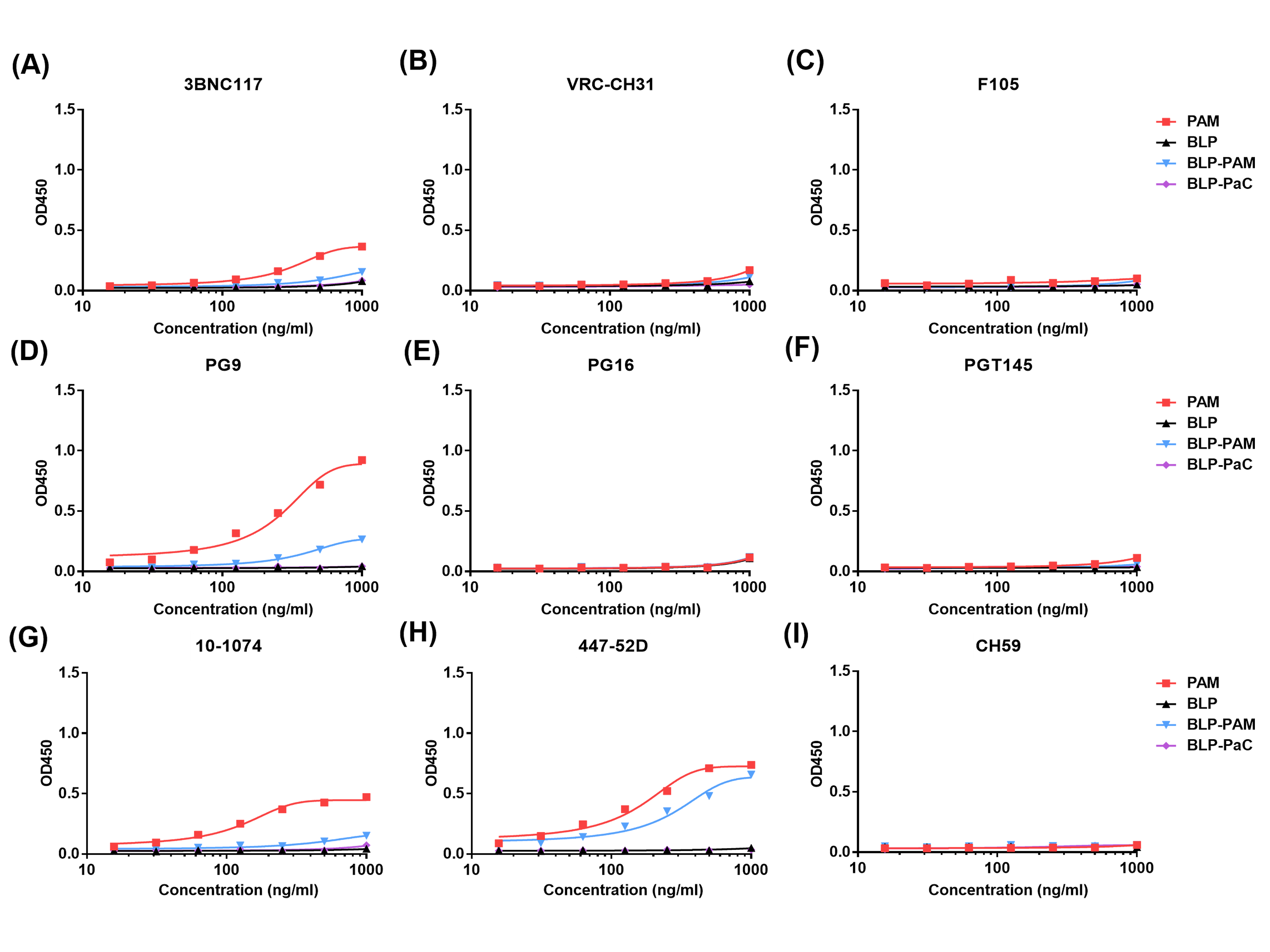


**Fig. S3.** Antigenicity analysis of PAM and BLP-PAM by ELISA.

1. C. Representative epitope exposing analysis of CD4bs (non-NAb of F105).
2. F. Representative binding curves of quaternary structure dependent bNAbs PG9, PG16, PGT145 targeting V1V2 Apex. G-I V3-glycan (10-1074), 447-52D (V3-loop), gp120 V2 (CH59). Note that the scales on the y-axes and x-axes vary from mAb to mAb. BLP and BLP-PaC were set as control.


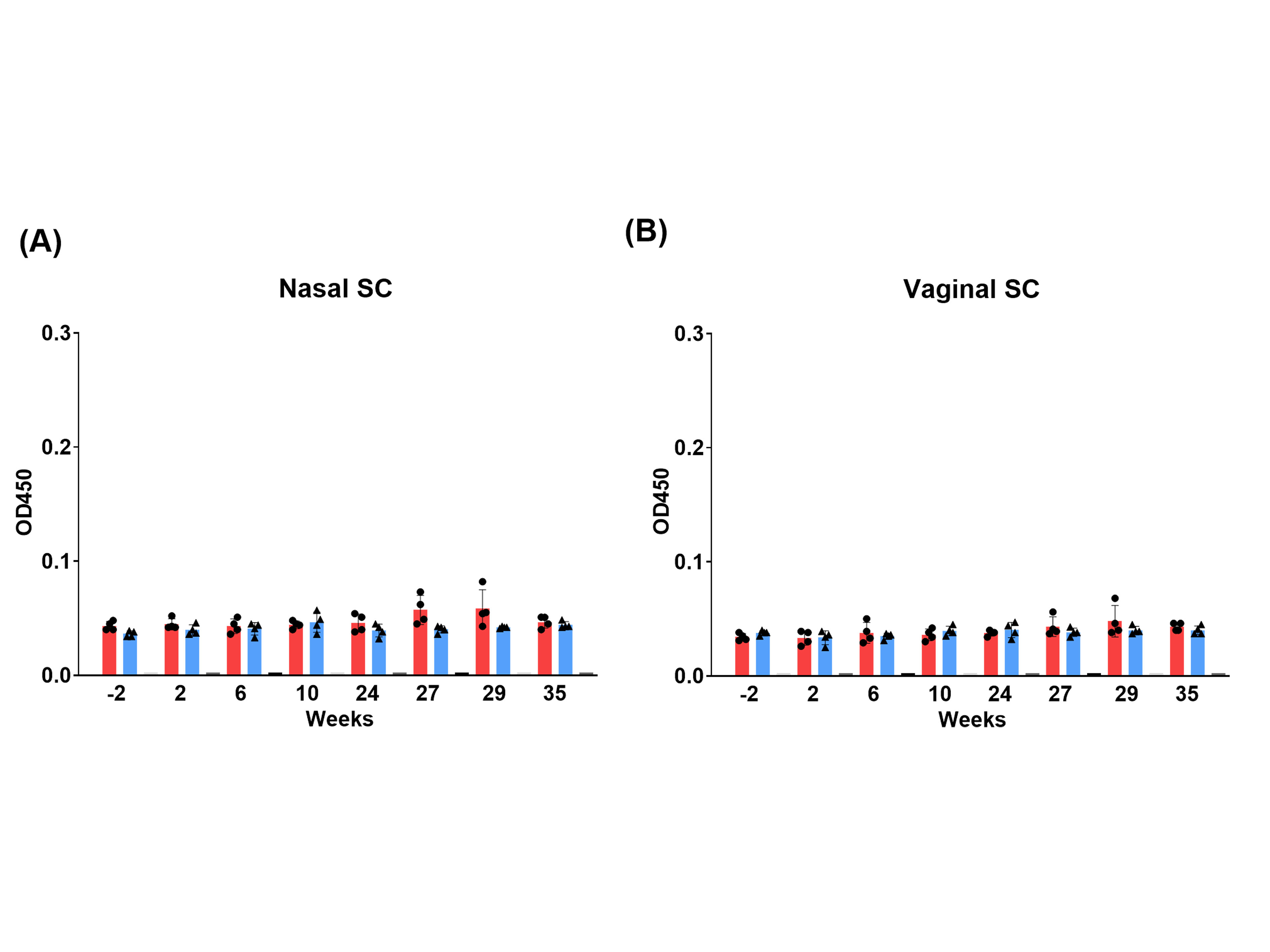


**Fig. S4. The mucosal immune responses in vaccinated rhesus macaques.**

A-B. Secretory component (SC) levels in nasal and vaginal washings between week -2 and week 35 measured by ELISA. Mean and standard deviation are indicated with lines and error bars.


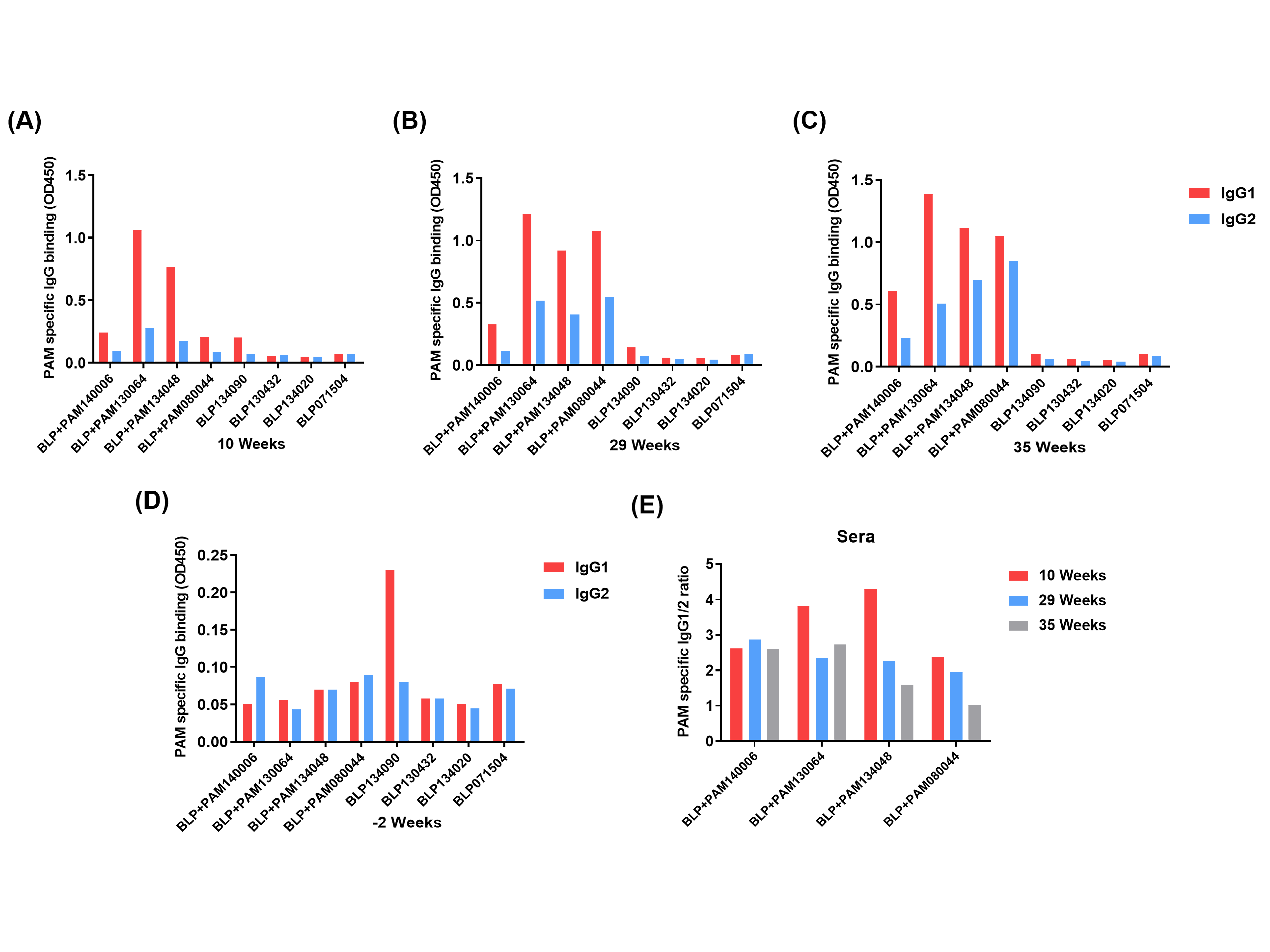


**Fig. S5. Isotype analysis of PAM specific antibody in sera.**

A-D. IgG isotype analysis in sera two weeks after pre-immunization and intramuscular immunization as measured by ELISA.

E. Ratio of IgG1/IgG2 in sera.


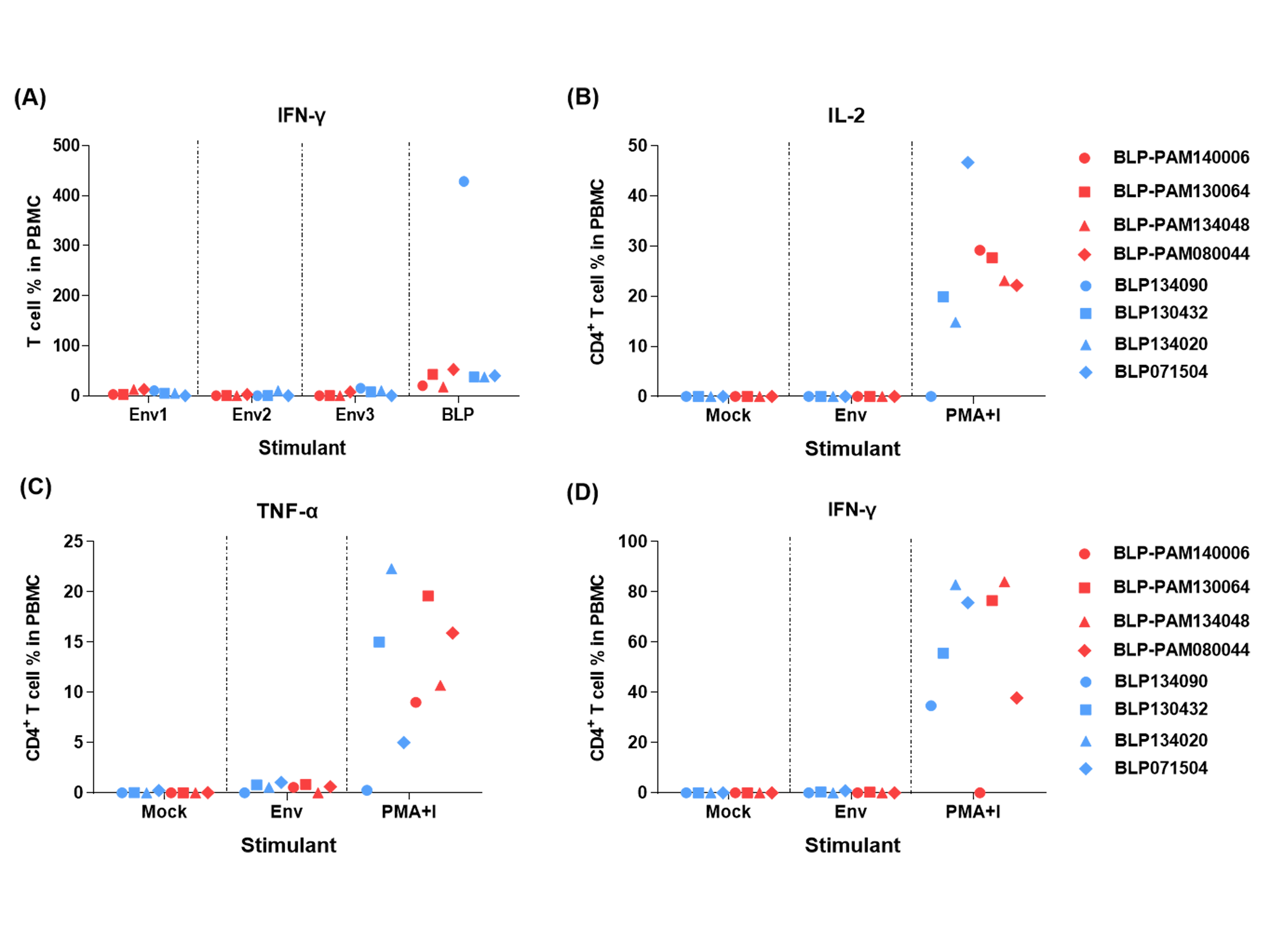


**Fig. S6. T cell immune responses in vaccinated rhesus macaques.**

1. IFN-γ^+^ secreting T cells per million PBMCs stimulated by Env peptide pools and “empty” BLP at 39 weeks PBMC measured by ELISPOT.

B-D. The percentages of IL-2^+^, TNF-α^+^ and IFN-γ^+^ CD4^+^ T cells in PBMC measured by FACS. PMA and ionomycin were used as control.


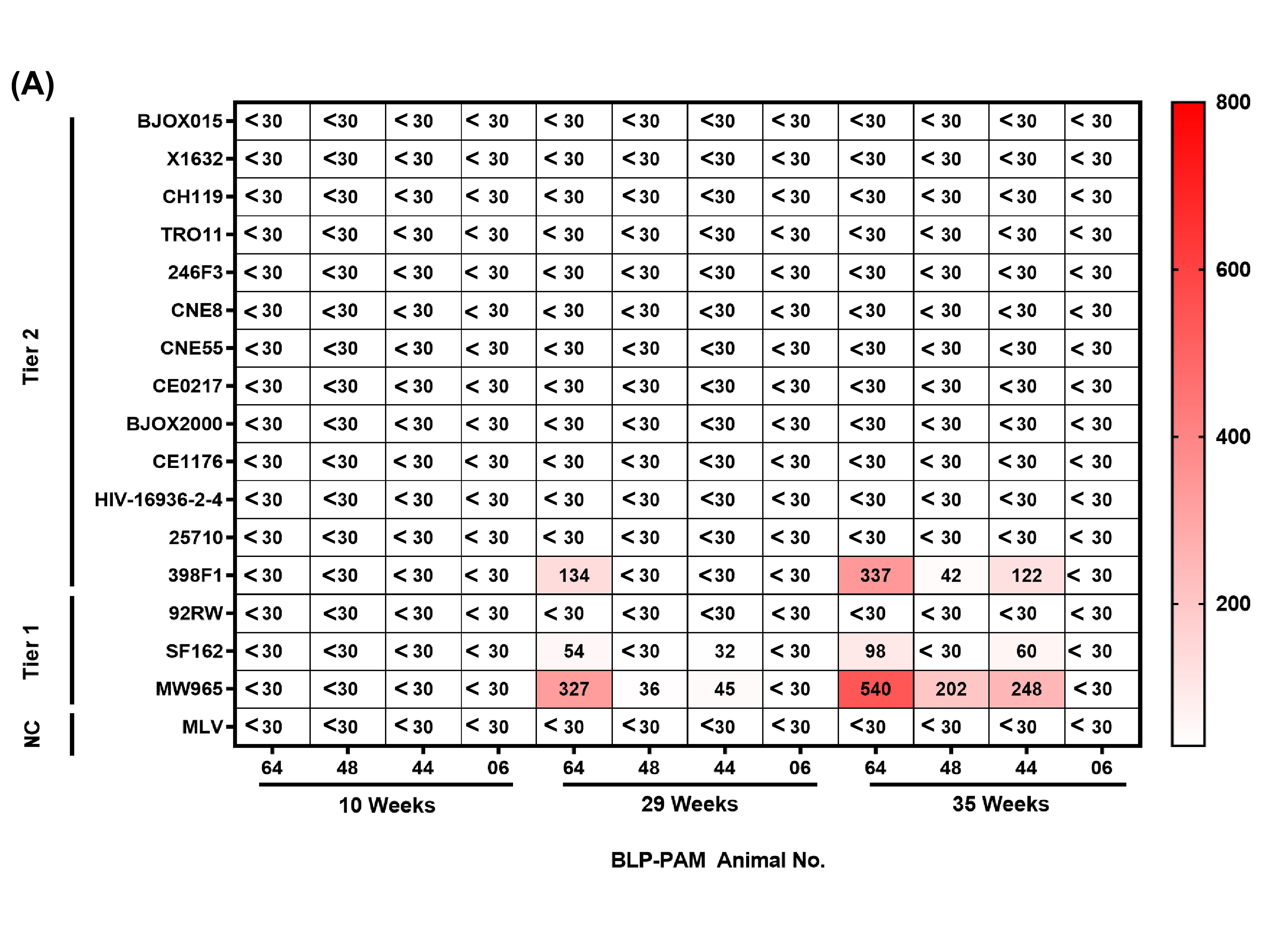

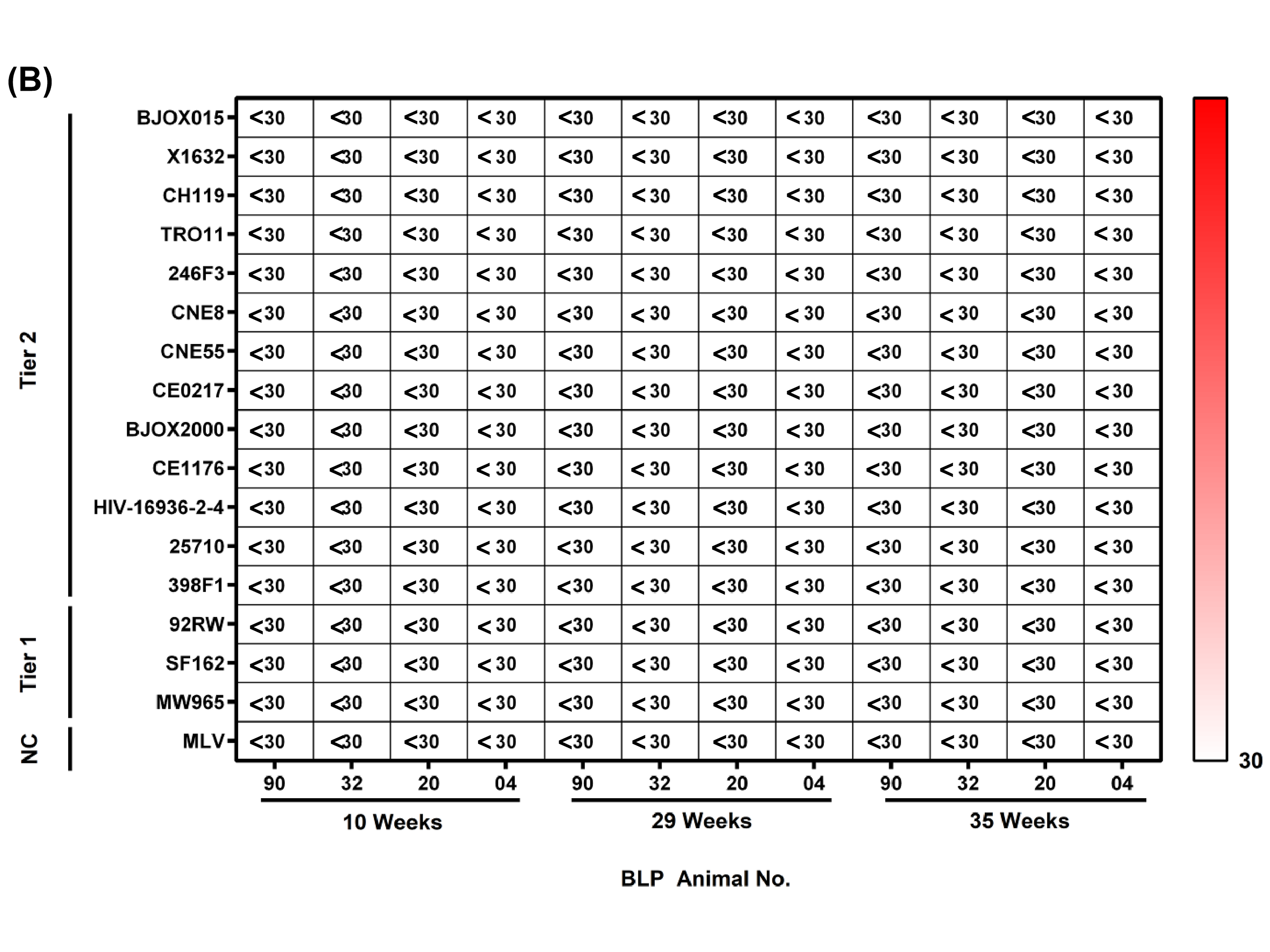


**Fig. S7. Neutralization titers (ID50) for sera from rhesus macaques tested against a panel of Env-pseudotyped viruses and mutants.**

1. Neutralization breath on Tier1 and Tier2 panel from BLP-PAM group sera samples.
2. Neutralization breath on Tier 1 and Tier 2 panel from BLP group serum samples.


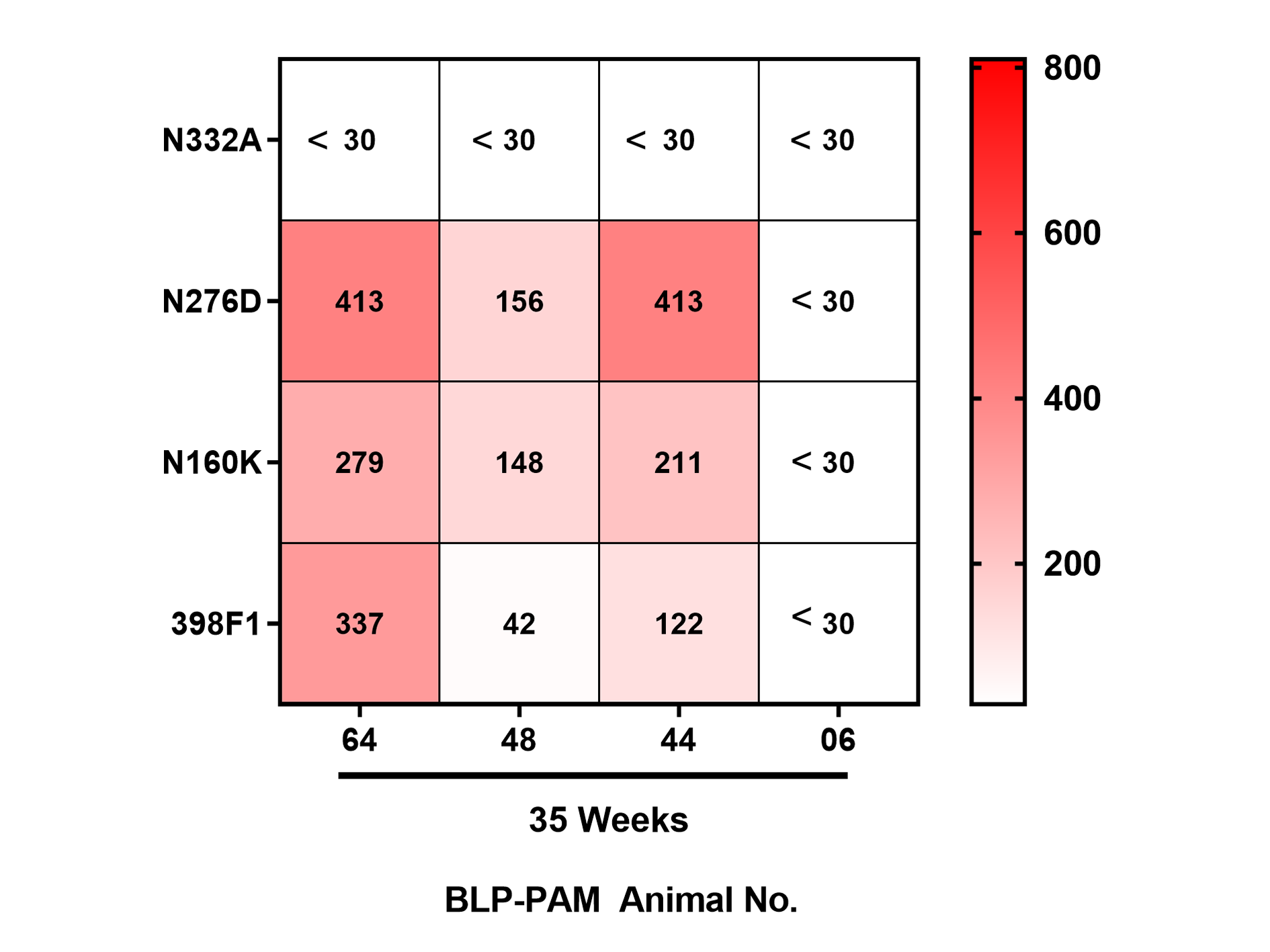


**Fig. S8.** Neutralization titers (ID50) from serum samples of rhesus macaques in BLP-PAM group tested against 398F1 mutants.


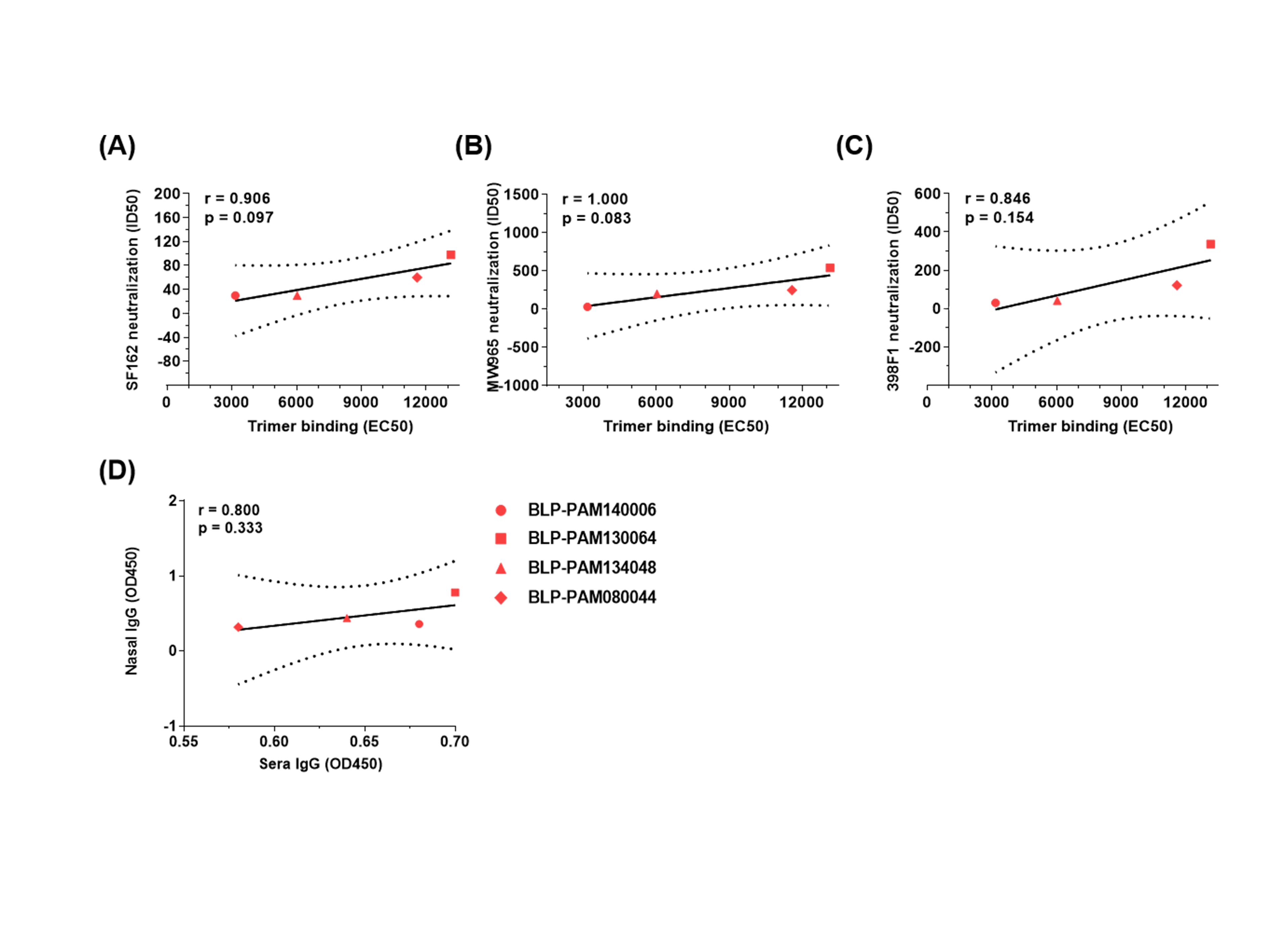


**Fig. S9.** Correlation analysis for antibody responses.

A-C. Correlation between binding antibody responses of sera to PAM and NAbs to Tier-1 (SF162 and MW965) Tier-2 viruses 398F1 at week 35.

D. Correlation between the IgG levels of sera and nasal washings at week 51.

The Pearson correlation coefficient, r, calculated using SPSS software version 22.0. for the respective correlations are given.


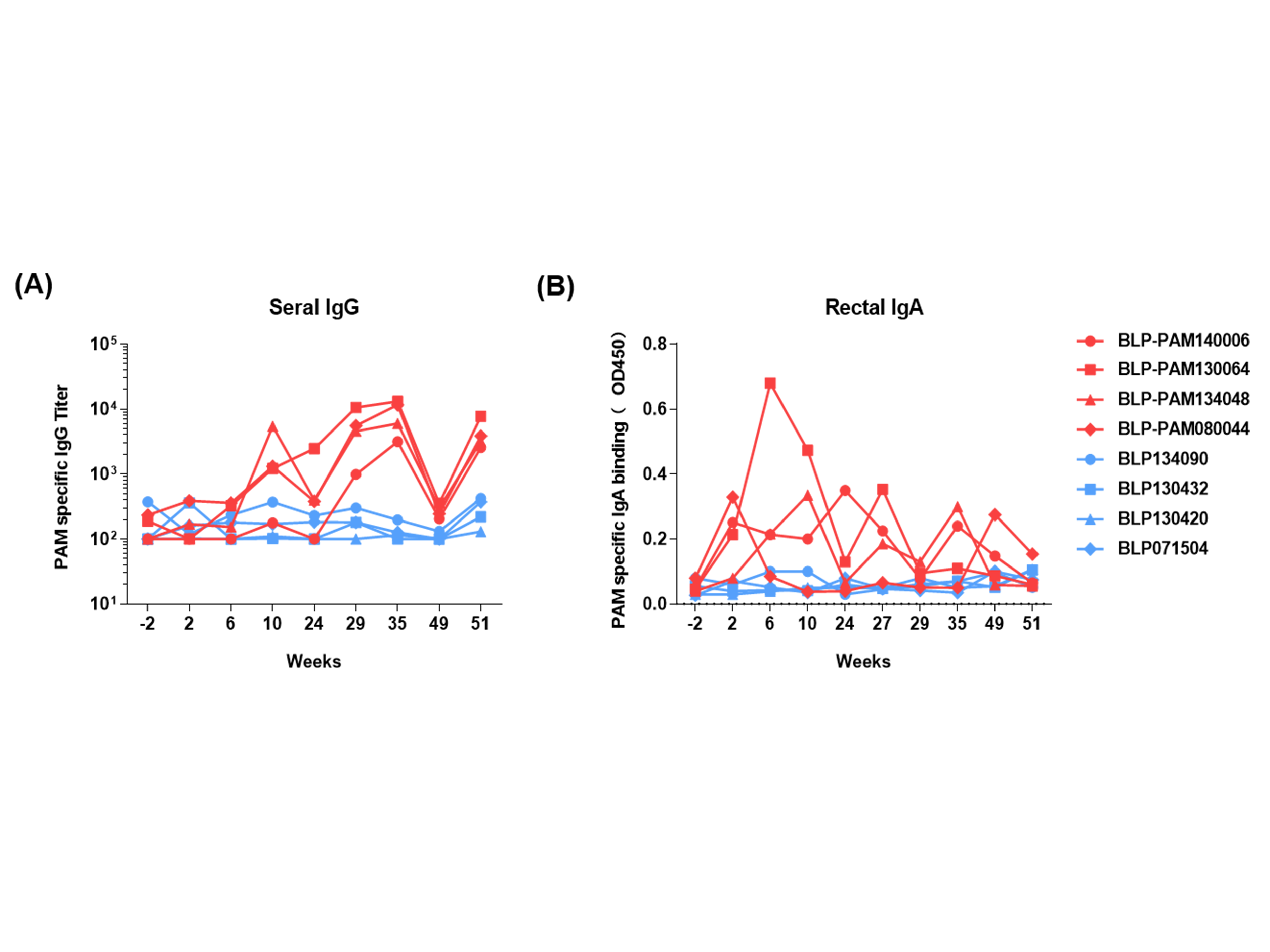


**Fig. S10. The humoral and mucosal responses of BLP-PAM in rhesus macaques.**

A. PAM specific IgG antibodies binding endpoint titers in sera measured by ELISA.

B. PAM specific IgA antibodies level in rectal samples.

**Supplementary Tables**

**Table S1 :** Kinetic analysis of Protan-gp120AE-MTQ and BG505 UFO binding to bNAbs and non-bNAbs by Bio-layer interferometry. Table shows Kon, Koff, KD.

| **Antigen** | **Antibody** | **K_on_(1/Ms)** | **K_off_ (1/s)** | **KD(M)** |
| --- | --- | --- | --- | --- |
| **gp120-PAM** | PG9 | 1.35×10^4^ | 1.37×10^-4^ | 1.01×10^-8^ |
|  | PG16 | 1.56×10^4^ | 1.26×10^-4^ | 1.36×10^-7^ |
|  | PGT145 | 5.43×10^4^ | 1.22×10^-3^ | 2.25×10^-8^ |
|  | 3BNC117 | 1.52×10^4^ | 1.00×10^-7^ | 1.00×10^-12^ |
|  | PGT121 | 1.45×10^4^ | 1.03×10^-4^ | 7.12×10^-9^ |
|  | 447-52D | 4.24×10^5^ | 5.89×10^-4^ | 1.39×10^-9^ |
| **BG505 UFO** | PG9 | 1.35×10^4^ | 1.37×10^-4^ | 1.01×10^-8^ |
|  | PG16 | 1.25×10^4^ | 1.28×10^-5^ | 1.02×10^-9^ |
|  | PGT145 | 5.40×10^4^ | 1.59×10^-3^ | 2.29×10^-8^ |
|  | 3BNC117 | 3.06×10^4^ | 4.14×10^-6^ | 1.36×10^-10^ |
|  | PGT121 | 3.67×10^4^ | 2.27×10^-5^ | 6.19×10^-10^ |
|  | 447-52D | 2.90×10^5^ | 4.43×10^-4^ | 1.53×10^-9^ |

**Table S2 :** Information of Chinese rhesus macaques used in this study.

| **Group** | **Animal ID** | **Sex** | **Weight(Kg)** | **Age(year)** | **Ad2 Nab** |
| --- | --- | --- | --- | --- | --- |
| BLP-PAM | 140006 | Female | 7.7 | 5 | <18 |
|  | 130064 | Female | 6.2 | 6 | <18 |
|  | 134048 | Female | 6.7 | 6 | <18 |
|  | 080044 | Female | 4.8 | 11 | <18 |
| BLP | 134090 | Female | 5 | 6 | <18 |
|  | 130432 | Female | 6.1 | 6 | <18 |
|  | 130420 | Female | 5.6 | 6 | <18 |
|  | 071504 | Female | 11.6 | 12 | <18 |

| **Antigen** | **Clone** | **Fluorophore** | **Supplier** | **Staining** | **Reactivity** |
| --- | --- | --- | --- | --- | --- |
| CD3 | SP34-2 | Pacific Blue | BD Biosciences | Surface | Human |
| CD4 | L200 | FITC | BD Biosciences | Surface | Human |
| IFN-γ | 4S.B3 | PE | BD Biosciences | Intracellular | Human |
| TNF-α | MAb11 | PE-Cy7 | BD Biosciences | Intracellular | Human |
| IL-2 | MQ1-17H12 | APC | BD Biosciences | Intracellular | Human |

**Table S3 :** Antibodies used for analytical flow cytometry in this study.
